# Crystal structures of [IrCl_2_(NHCHPh)((dppm)(C(N_2_dppm))-κ^3^ *P*,*C*,*P*′)]Cl·5.5MeCN and [IrI(NHCHPh)(((dppm)C(N_2_))-κ^2^ *P*,*C*)(dppm-κ^2^ *P*,*P*′)]I(I_3_)·0.5I_2_·MeOH·0.5CH_2_Cl_2_: triazene fragmentation in a PCN pincer iridium complex

**DOI:** 10.1107/S2056989019000136

**Published:** 2019-01-08

**Authors:** Bettina Pauer, Gabriel Julian Partl, Stefan Oberparleiter, Walter Schuh, Holger Kopacka, Klaus Wurst, Paul Peringer

**Affiliations:** a Institute of General, Inorganic and Theoretical Chemistry, University of Innsbruck, A-6020 Innsbruck, Austria

**Keywords:** crystal structure, carbodi­phospho­rane, divalent carbon, triazene, fragmentation, diazo­methyl­ene­phosphor­ane, diazo­phosphor­ane, nitrene, iridium(III)

## Abstract

The structure of [IrCl_2_(C_58_H_51_N_3_P_4_)]Cl·5.5CH_3_CN or [IrCl_2_(NHCHPh)(((dppm)C(N_2_dppm))*-*κ*^3^P,C,P*)]Cl·5.5CH_3_CN [**3**, dppm = bis­(di­phenyl­phos­phino)methane; systematic name: di­chlorido(1,1,3,3,7,7,9,9-octa­phenyl-4,5-di­aza-1,3λ^5^,7λ^4^,9-tetra­phosphanona-3,5-dien-6-yl-κ^2^ *P* ^1^,*P* ^9^)­(phenyl­methanimine-κ*N*)iridium(III) chloride aceto­nitrile hemihendeca­solvate], resulting from an oxygen-mediated cleavage of a triazeneyl­idene­phospho­rane ligand producing a diazo­methyl­ene­phospho­rane and a nitrene moiety, which in turn rearrange *via* a Staudinger reaction and a 1,2-hydride shift to the first title complex, involves a six-coordinate Ir^III^ complex cation coordinated by a *facial* PCP pincer ligand, a benzaldimine and two chlorido ligands. The pincer system features a five- and a seven-membered ring, with the central divalent carbon of the PCP pincer ligand being connected to a phosphine and a diazo­phospho­rane. The chlorido ligands are positioned *trans* to the central carbon atom and to the phospho­rus donor of the seven-membered ring of the pincer system, respectively. A chloride ion serves as counter-ion for the monocationic complex. The structure of [IrI(C_26_H_22_N_2_P_2_)(C_26_H_22_P_2_)(C_6_H_7_N)]I(I_3_)·0.5I_2_·CH_3_OH·0.5CH_2_Cl_2_ or [IrI(NHCHPh)((dppm)C(N_2_)-κ*^2^P,C*)(dppm*-*κ*^2^P,P′*)]I(I_3_)·0.5I_2_·CH_3_OH·0.5CH_2_Cl_2_ {**4**, systematic name: (4-diazo-1,1,3,3,-tetra­phenyl-1,3λ^4^-diphosphabutan-4-yl-κ*P* ^1^)iodido[methyl­enebis(di­phenyl­phosphine)-κ^2^ *P*,*P*′](phenyl­methanimine-κ*N*)iridium(III) iodide–triiodide–di­chloro­methane–iodine–methanol (2/2/1/1/2)}, accessed *via* treatment of the triazeneyl­idene­phospho­rane complex [Ir((BnN_3_)C(dppm)-κ*^3^P,C,N*)(dppm-κ*^2^P,P*′)]Cl with hydro­iodic acid, consists of a dicationic six-coordinate Ir^III^ complex, coordinated by a bidentate diazo­methyl­ene­phospho­rane, a benzaldimine, a chelating dppm moiety and an iodido ligand. The phospho­rus atoms of the chelating dppm are *trans* to the central carbon atom of the diazo­methyl­ene­phospho­rane and the iodide ligand, respectively. Both an iodide and a triiodide moiety function as counter-ions. The aceto­nitrile solvent mol­ecules in **3** are severely disordered in position and occupation. In **4**, the I_3_^−^ anion is positionally disordered (ratio roughly 1:1), as is the I^−^ anion with a ratio of 9:1. The di­chloro­methane solvent mol­ecule lies near a twofold rotation axis (disorder) and was refined with an occupancy of 0.5. Another disorder occurs for the solvent methanol with a 1:1 ratio.

## Abstract 2

In this communication, two compounds and their respective crystal structures, obtained *via* fragmentation of the triazene moiety in a PCN pincer iridium complex, are discussed. One showcases a novel (dppm)C(N_2_dppm) PCP pincer, the other contains a (dppm)C(N_2_) diazo­methyl­ene­phospho­rane moiety.

## Chemical context   

A peculiarity of triazenes is that their N—N bonds are comparatively easily cleaved. This may result, other than N_2_ extrusion reactions, in diazo­nium and quaternary ammonium moieties, in diazo compounds and amines (Baumgarten, 1967 ▸; Schroen & Bräse, 2005 ▸), or in diazo compounds and amides (Myers & Raines, 2009 ▸) depending on the triazene substitution pattern. By taking advantage of their reactivity, the transformation of organic azides into diazo compounds *via* triazene inter­mediates has developed into a broad synthetic route to diazo compounds (Myers & Raines, 2009 ▸).
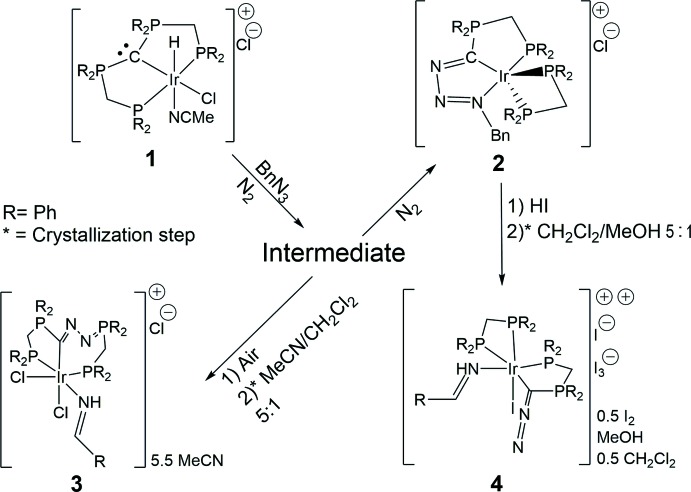


In this contribution, we describe the fragmentation of a triazene into diazo and nitrene parts in the coordination sphere of iridium. Recently, we reported on the synthesis of [Ir((4-Cl-C_6_H_4_N_3_)C(dppm)-κ*^3^P,C,N*)(dppm*-*κ*^2^P,P′*)]Cl *via* treatment of [Ir(Cl)(H)(MeCN)(C(dppm)_2_-κ^3^ *P,C,P*)] (**1**) with 1-azido-4-chloro­benzene under an inert atmosphere (Partl *et al.*, 2019 ▸). The triazeneyl­idene­phospho­rane (4-Cl-C_6_H_4_N_3_)C(dppm) unit of this compound is generated *via* substitution of one phosphine moiety of the carbodi­phospho­rane (CDP) C(dppm)_2_ of **1** for the organic azide. This substitution reaction results in the formation of a labile Ir^I^ inter­mediate, whose coordination sphere features the PCN pincer ligand (4-Cl-C_6_H_4_N_3_)C(dppm) and a monodentate dppm (Partl *et al.*, 2019 ▸). Analogously, a related inter­mediate and product (**2**) are created by using benzyl azide, rather than 1-azido-4-chloro­benzene, under an inert atmosphere.

When the inter­mediate (in the case of benzyl azide) is brought into contact with air, pale-yellow crystals of compound **3** separate within a few hours. It contains a novel PCP pincer system involving one seven- and one five-membered ring. The difference to the PCP pincer ligand of the starting complex **1** is that the pincer of **3** has an N_2_ moiety inserted into one P—C bond of the CDP functionality of C(dppm)_2_. Regarding the reaction mechanism, we propose that first, the Ir^I^ center of the inter­mediate is oxidized by atmospheric oxygen. This is presumably followed by a homolytic cleavage of the N2—N3 bond (numbering according to the crystal structure of **3**) of (BnN_3_)C(dppm), producing the diazo­methyl­ene­phospho­rane (dppm)C(N_2_) involving N1 and N2, and a benzyl­nitrene moiety containing N3.

*Via* an intra­molecular Staudinger reaction (Staudinger & Meyer, 1919*a* ▸,*b* ▸) of the diazo functionality of (dppm)C(N_2_) with the pendent phosphine of the monodentate dppm ligand, the phosphazine (dppm)C(N_2_dppm) is formed and subsequently acts as PCP pincer ligand. In this ligand, the central divalent carbon (Petz & Frenking, 2010 ▸) of (dppm)C(N_2_dppm) connects to one tertiary phosphine of the dppm unit, and to a diazo­phospho­rane (Murahashi *et al.*, 2005 ▸). The benzyl­nitrene undergoes a 1,2-hydride shift, thus producing a benzaldimine moiety that remains in the coord­in­ation sphere of iridium. In this context, it is very noteworthy that the scission of the N1—N2 bond occurs in the course of the aforementioned transformation of organic azides into diazo compounds *via* triazenes (Myers & Raines, 2009 ▸).

In a related fragmentation reaction, compound **4** was obtained through treatment of [Ir((BnN_3_)C(dppm)-κ*^3^P,C,N*)(dppm-κ*^2^P,P′*)]Cl (**2**) with hydro­iodic acid. It is apparent that a rupture of the N1—N2 bond (numbering as in the structure of **4**) of (BnN_3_)C(dppm) occurred again, resulting in the formation of a diazo­methyl­ene­phospho­rane (dppm)C(N_2_) and a benzyl­nitrene part. However, in this case, the diazo functionality remains unchanged, since in contrast to the formation of **3**, no free phosphine functionality is available. The benzyl­nitrene unit again undergoes a 1,2-hydride shift and, as a benzaldimine, coordinates to the Ir metal center.

The resonance structures of diazo compounds include ylene and ylide structures, as is the case for phospho­rus ylides. The diazo­methyl­ene­phospho­rane (dppm)C(N_2_) moiety contains a central divalent carbon (Petz & Frenking, 2010 ▸), to which a phosphine and an N_2_ donor are formally attached and which may be considered as a mixed double ylide (Petz & Frenking, 2010 ▸). Related compounds of the type C(P*X*(NMe_2_)_2_)(N_2_), *X* = Cl, Br, were obtained by addition of C*X*_4_ to P((NMe_2_)_2_)(CH(N_2_)) (Sotiropoulos *et al.*, 1987 ▸).

## Structural commentary   

**Figure 1:**
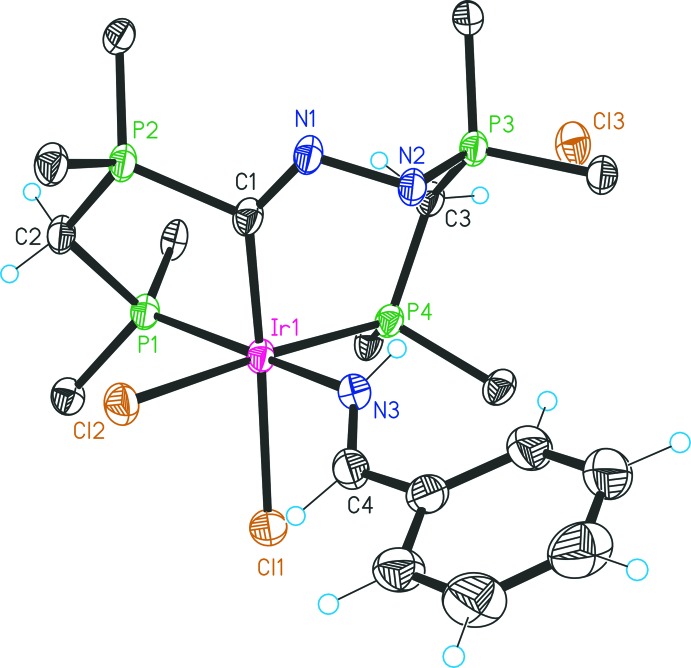
A view of the mol­ecular structure of the cation of compound **3**, with displacement ellipsoids drawn at the 30% probability level and atom labelling. Only the *ipso* carbon atoms of the dppm phenyl groups are shown, and solvate mol­ecules have been omitted for clarity.

The structure of **3** (Fig. 1 ▸) shows a six-coordinate monocationic Ir^III^ complex and one chloride counter-ion. The asymmetric unit contains one formula unit and 5.5 mol­ecules of MeCN. Selected bond lengths and bond angles of **3** are given in Table 1 ▸. The most significant intra­molecular inter­actions are listed in Table 2 ▸. The iridium center is coordinated by the *facial* PCP pincer system, which involves one seven-membered IrC(N_2_dppm) ring and one five-membered IrC(dppm) ring. A benzaldimine ligand is positioned *trans* to the phospho­rus donor of the five-membered ring, the remaining two coordination sites being occupied by chlorido ligands *cis* to each other. The deviations of the angles C1—Ir1—Cl1 = 170.06 (13)° and N3—Ir1—P1 = 169.02 (11)° from a regular octa­hedral geometry indicate some strain in the pincer system. Both the N1—C1 bond length [1.280 (5) Å] and the N1—N2 bond length [1.445 (5) Å] are typical for a C=N double bond and an N—N single bond, respectively. The P3—N2 bond length [1.586 (4) Å] is in the range of P=N double bonds observed for imino­phospho­ranes (Ireland *et al.*, 2010 ▸; Peng *et al.*, 2011 ▸; Sun *et al.*, 2011 ▸). Corresponding bond lengths in other phosphazene systems exhibit values of 1.62–1.64 Å for P—N, 1.36–1.39 Å for N—N and 1.31 Å for C—N. (Bethell *et al.*, 1992 ▸; Supurgibekov *et al.*, 2011 ▸; Galina *et al.*, 2013 ▸; Nikolaev *et al.*, 2016 ▸). The P2—C1 bond length [1.836 (4) Å] indicates a single bond. The environment around C1 is strictly planar (sum of the angles amounts to 359.7°). Examination of the C4—N3 bond length within the benzaldimine ligand [1.270 (6) Å] indicates a double bond and is almost identical to that observed in compound **4** [1.267 (8) Å] and a previously reported iridium benzaldimine complex [1.260 (6) Å] involving a phospho­rus donor atom *trans* to the benzaldimine nitro­gen donor (Albertin *et al.*, 2008 ▸). The most striking intra­molecular inter­action of **3** is the hydrogen bond N3—H3*N*⋯N2 [H⋯A 2.15 (5) Å, *D*—H⋯*A* 138 (4)°], while other intra­molecular inter­actions involve atoms N1 and Cl1 and the various phenyl rings (Table 2 ▸).

**Figure 2:**
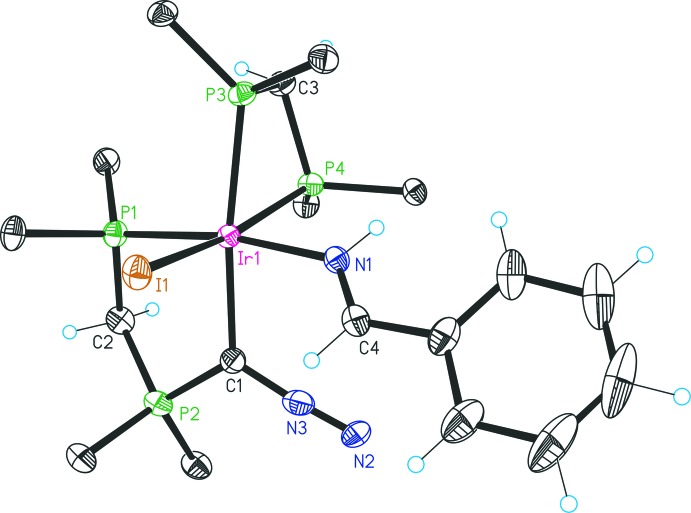
A view of the mol­ecular structure of the cation of compound **4**, with displacement ellipsoids drawn at the 30% probability level and atom labelling. Only the *ipso* carbon atoms of the dppm phenyl groups are shown, the anions and solvate mol­ecules have been omitted for clarity.

The structure of **4** (Fig. 2 ▸) consists of a six-coordinate dicationic Ir^III^ complex, one iodide and one triiodide counter-ion. The asymmetric unit contains one half mol­ecule of di­chloro­methane and iodine and one mol­ecule of methanol. Selected bond lengths and angles of **4** are summarized in Table 1 ▸. The most significant intra­molecular inter­actions are listed in Table 3 ▸. The iridium center is coordinated by the bidentate ligand (dppm)C(N_2_), which forms a five-membered chelate ring *via* one C and one P donor atom. A four-membered ring is formed by a bidentate dppm ligand and is oriented perpendicular to the plane of the five-membered ring with one phospho­rus donor *trans* to the carbon donor of the five-membered ring. The benzaldimine ligand is located *trans* to the phospho­rus donor of the five-membered ring, the sixth coordination site is occupied by an iodido ligand. Deviations from the octa­hedral symmetry around the Ir center are mainly due to the strained four-membered ring [P4—Ir1—P3 = 70.80 (5)°] with consequences for the bond angles P4—Ir1–I1 [165.12 (4)°] and C1—Ir1—P3 [170.90 (17)°]. The C1—Ir1—P1 bond angle of the five-membered ring is 84.37 (17)°. The environment around the ylidic carbon C1 is trigonal planar, with the bond angle N3—C1—P2 exhibiting the largest deviation from a regular symmetry [114.8 (5)°]. Both the N3—N2 [1.095 (9) Å] and the N3—C1 [1.305 (9) Å] bond lengths are slightly shorter, compared to the corresponding mean values of ten previously reported structures of diazo compounds [1.121 and 1.323 Å, respectively; Cambridge Structural Database (Groom *et al.*, 2016 ▸)]. The P2—C1 bond length [1.753 (6) Å] is shorter than a P—C single bond, but is similar to phospho­rus ylide complexes of iridium (Campos *et al.*, 2013 ▸). The most striking intra­molecular inter­action of **4** is an N—H⋯π inter­action N1—H1*N*⋯*Cg* (*Cg* being the centroid of phenyl ring C407–C412, H⋯*Cg* 2.87 (5) Å, N—H⋯*Cg* 145 (4)°); see Table 3 ▸. Other intra­molecular inter­actions involve atoms N2 and I1 (C408—H408⋯N2 and C4—H4⋯I1) given in Table 3 ▸.

## Supra­molecular features   

**Figure 3:**
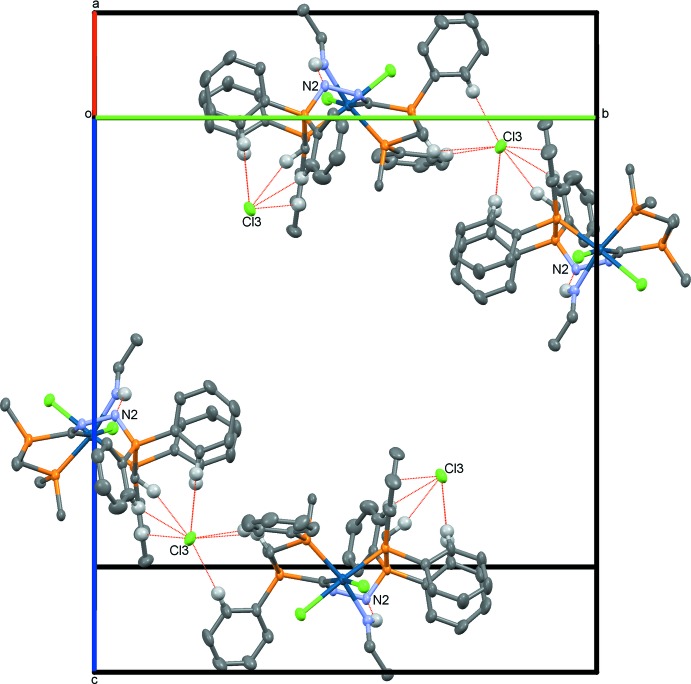
A view along the a* axis of the crystal packing of **3**, highlighting some of the intra- and inter­molecular inter­actions. For clarity, solvate mol­ecules and non-involved H atoms have been omitted, and for uninvolved phenyl moieties, only the *ipso* carbon atoms are displayed.

In the crystal of **3**, the cationic complexes are inter­connected through the chloride anions *via* essentially C—H⋯Cl3 hydrogen bonds. The most significant hydrogen-bonding inter­actions are given in Table 2 ▸. Of these, two stem from phenyl groups and one from a methyl­ene group of the PCP pincer’s dppmN_2_ part (H3*A*⋯Cl3 2.63 Å). It is worth mentioning that such inter­actions are frequently observed in dppm and related ligands (Jones & Ahrens, 1998 ▸). A graphical representation of these inter­actions is given in Fig. 3 ▸. Effectively, the C—H⋯Cl3 hydrogen bonds link the cationic complexes, forming chains propagating along the *b*-axis direction.

**Figure 4:**
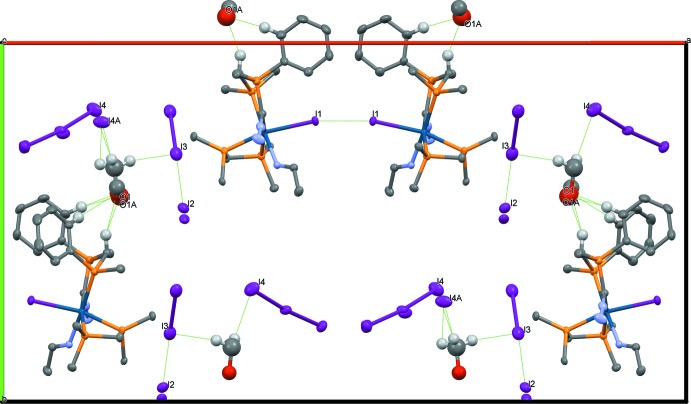
A view along the *c* axis of the crystal ordering of **4**, highlighting some of the inter­molecular inter­actions. For clarity, uninvolved solvate mol­ecules and H atoms have been omitted, and for non-involved phenyl groups, only the *ipso* carbon atoms are displayed.

In the crystal of **4**, solvent inter­actions are centered around atoms O1 and O1*A* of the methanol mol­ecule *viz*. two phenyl protons (H112⋯O1 2.55 and H212⋯O1*A* 2.31 Å) and one dppm methyl­ene moiety (H2*A*⋯O1 2.25 and H2*A*⋯O1*A* 2.28 Å) (Jones & Ahrens, 1998 ▸) attach to the oxygen atom of the disordered methanol group *via* hydrogen bonding (Fig. 4 ▸). These and the other most significant inter­molecular inter­actions are given in Table 3 ▸. Together with C—H⋯I2(I2*A*) hydrogen bonds and a C—H⋯π inter­action, a supra­molecular layer is formed lying parallel to the *bc* plane (Table 3 ▸). The iodine hemisolvate coordinates to the iodide anion [I2⋯I3 3.443 (1) Å]. As far as true inter­molecular inter­actions go, iridium-bound iodide moieties appear to bind to each other through weak halogen–halogen inter­actions [I1⋯I1′ 3.890 (1) Å]. Fig. 4 ▸ displays these inter­actions in graphical fashion.

## Synthesis and crystallization   

The syntheses of the title compounds are summarized in the reaction scheme. ^1^H, ^13^C and ^31^P NMR spectra were recorded on a Bruker DPX 300 NMR spectrometer (300 MHz) and were referenced against ^13^C/^1^H solvent peaks or an external 85% H_3_PO_4_ standard, respectively. The phospho­rus atoms in the NMR data are labelled in the same way as in the figures.

**Synthesis and crystallization of complex 3:** A mixture of [IrCl(cod)]_2_ (8.5 mg; 0.0125 mmol) and [CH(dppm)_2_]Cl (Reitsamer *et al.*, 2012 ▸) (20.5 mg; 0.025 mmol) was placed under an inert atmosphere (N_2_), dissolved in acetone (0.6 ml) and stirred for 3 h. The resulting white precipitate of [IrCl_2_H(C(dppm)_2_)] (Partl *et al.*, 2018 ▸) was separated via centrifugation and deca­ntation. To it, MeCN (0.5 mL) and a solution of BnN_3_ in CH_2_Cl_2_ (0.1 ml; 0.5 mol L^−1^; 0.050 mmol) were added. After stirring for 1 min, the deep-purple solution was stirred for 2 h under atmospheric conditions, resulting in the slow precipitation of a white product. Colourless to pale-yellow prismatic crystals of **3** were obtained by allowing the purple inter­mediate solution to stand overnight under ambient conditions.

^31^P{^1^H}-NMR (CHCl_3_/MeOH 1:1): *δ* = 0.7 (P1, *dd*, *J*_P1P2_ = 30.7, *J*_P1P4_ = 14.0 Hz); 16.9 (P2, *ddd*, *J*_P2P3_ = 13.6 Hz, *J*_P2P4_ = 3.7 Hz); 6.9 (P3, *d*); −43.1 (P4, *dd*) ppm. ^13^C{^1^H}-NMR (CHCl_3_/MeOH 1:1): *δ* =157.9 (C1, *dd*, *J*_C1P2_ = 47.4, *J*_C1P3_ = 16.5 Hz) ppm.

**Synthesis and crystallization of complex 4:** Under an inert atmosphere, a mixture of [IrCl(cod)]_2_ (8.5 mg; 0.0125 mmol), [CH(dppm)_2_]Cl (20.5 mg; 0.025 mmol) (Reitsamer *et al.*, 2012 ▸) and MeCN (0.1 ml) was allowed to stir for 3 min. While stirring, MeOH (0.5 ml) and BnN_3_ in CH_2_Cl_2_ (0.1 ml; 0.5 mol/L; 0.050 mmol) were added. After heating to 333 K for 15 min, the volatiles were removed *in vacuo*. The residue was dissolved in CH_2_Cl_2_ and hydro­iodic acid (0.030 ml, 0.31 mmol, 67%) was added whilst stirring. The orange–brown precipitate that formed slowly was separated, washed with water and dried *in vacuo*. A solution of the residue in CH_2_Cl_2_/MeOH 2:1 (0.6 ml) qu­anti­tatively contained an unidentified inter­mediate, which transformed to the product within 1 h. Red prismatic crystals of **4** formed within a few hours, when a solution of the inter­mediate in CH_2_Cl_2_/MeOH (5:1) was kept at 254 K for 24 h and subsequently warmed to room temperature.

^31^P{^1^H}-NMR (CH_2_Cl_2_/MeOH 2:1): *δ* = −15.8 (P1, *ddd*, *J*_P1P2_ = 16.8, *J*_P1P3_ = 15.3, *J*_P1P4_ = 13.8 Hz); 37.8 (P2, *d*); −72.1 (P3, *dd*, *J*_P3P4_ = 28.3 Hz); −62.8 (P4, *dd*) ppm. ^13^C{^1^H}-NMR (CH_2_Cl_2_/MeOH 2:1): *δ* = −3.0 (C1, *ddd*, *J*_C1P2_ = 68.2, *J*_C1P3_ = 105.2, *J*_C1P4_ = 4.6 Hz) ppm.

## Refinement   

Crystal data, data collection and structure refinement details are summarized in Table 4 ▸. The aceto­nitrile solvent mol­ecules in the crystal lattice of **3** are severely disordered in position and occupation. At least 5.5 mol­ecules in the asymmetric units were refined. Occupation values were varied to give a reasonable isotropic displacement factor. All C- and N-atoms of solvent mol­ecules were refined isotropically with bond restraints, the hydrogen atoms were omitted. The proton on N3 was freely refined.

The hydrogen atom at N1 of **4** was found and refined with a bond restraint of 0.87 (2) Å. The I_3_^−^ anion (I4–I6) is positionally disordered (ratio roughly 1:1), as is the I^−^ anion with a ratio I2:I2*A* of 9:1. The di­chloro­methane solvent mol­ecule lies near a twofold rotation axis (disorder) and was refined with an occupancy of 0.5. Another disorder occurs for the solvent methanol with a ratio of 1:1. The C and O atoms of methanol were refined isotropically with bond restraints of 1.40 Å. The hydrogen atoms of methanol were calculated, those of di­chloro­methane omitted. All other H atoms were positioned geometrically (C—H = 0.94–0.98 Å) and refined as riding with *U*_iso_(H) = 1.2-1.5 *U*_eq_(C).

## Supplementary Material

Crystal structure: contains datablock(s) global, 3, 4. DOI: 10.1107/S2056989019000136/su5470sup1.cif

Structure factors: contains datablock(s) 3. DOI: 10.1107/S2056989019000136/su54703sup2.hkl

Structure factors: contains datablock(s) 4. DOI: 10.1107/S2056989019000136/su54704sup3.hkl

CCDC references: 1885936, 1885935

Additional supporting information:  crystallographic information; 3D view; checkCIF report

## Figures and Tables

**Table 1:** Selected bond distances (Å) and angles (°) for compounds **3** and **4**

**3**		**4**	
Ir1—C1	2.044 (4)	Ir1—C1	2.150 (6)
Ir1—N3	2.077 (4)	Ir1—N1	2.107 (5)
Ir1—P1	2.3090 (12)	Ir1—P1	2.3468 (16)
Ir1—P4	2.3151 (11)	Ir1—P4	2.3241 (15)
Ir1—Cl1	2.4595 (12)	Ir1—P3	2.3536 (15)
Ir1—Cl2	2.4094 (11)	Ir1—I1	2.7206 (5)
P2—C1	1.837 (4)	P2—C1	1.753 (6)
N1—C1	1.280 (5)	N3—C1	1.305 (9)
N1—N2	1.445 (5)	N2—N3	1.095 (9)
N3—C4	1.270 (6)	N1—C4	1.267 (8)
P3—N2	1.586 (4)		
			
N3—Ir1—P1	169.02 (11)	N1—Ir1—P1	170.93 (14)
P4—Ir1—Cl2	176.55 (4)	P4—Ir1—I1	165.12 (4)
C1—Ir1—Cl1	170.06 (13)	C1—Ir1—P3	170.90 (18)
C1—Ir1—P1	86.06 (12)	C1—Ir1—P1	84.37 (17)
			
N1—C1—Ir1	134.2 (3)	P4—Ir1—P3	70.80 (5)
N1—C1—P2	109.1 (3)	N3—C1—P2	114.8 (5)
P2—C1—Ir1	116.4 (2)	N3—C1—Ir1	121.4 (5)
N1—N2—P3	110.3 (3)	P2—C1—Ir1	122.9 (3)
C1—N1—N2	117.5 (3)	N2—N3—C1	175.8 (7)

**Table 2:** Hydrogen-bond geometry (Å, °) for **3** [Chem scheme1]

*D*—H⋯*A*	*D*—H	H⋯*A*	*D*⋯*A*	*D*—H⋯*A*
N3—H3*N*⋯N2	0.82 (5)	2.15 (5)	2.807 (6)	138 (4)
C208—H208⋯N1	0.93	2.41	3.088 (7)	130
C402—H402⋯N3	0.93	2.56	3.120 (6)	119
C102—H102⋯Cl1	0.93	2.71	3.329 (5)	125
C402—H402⋯Cl1	0.93	2.66	3.428 (5)	140
C412—H412⋯Cl1	0.93	2.60	3.440 (5)	151
C3—H3*A*⋯Cl3	0.97	2.63	3.563 (5)	162
C105—H105⋯Cl3^i^	0.93	2.69	3.586 (7)	162
C408—H408⋯Cl3	0.93	2.82	3.533 (6)	134

Symmetry code: (i) 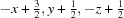.

**Table 3:** Hydrogen-bond geometry (Å, °) for **4** [Chem scheme1] *Cg* is the centroid of the C407–C412 ring.

*D*—H⋯*A*	*D*—H	H⋯*A*	*D*⋯*A*	*D*—H⋯*A*
N1—H1*N*⋯*Cg*	0.86 (2)	2.87 (5)	3.608 (6)	145 (4)
C408—H408⋯N2	0.94	2.57	3.35 (1)	141
C4—H4⋯I1	0.94	2.98	3.45 (1)	112
C2—H2*A*⋯O1	0.98	2.25	3.19 (2)	160
C2—H2*A*⋯O1*A*	0.98	2.28	3.11 (3)	142
C112—H112⋯O1	0.94	2.55	3.41 (3)	154
C212—H212⋯O1*A*	0.94	2.31	3.20 (4)	159
C3—H3*B*⋯I2	0.98	3.02	3.89 (1)	149
C106—H106⋯I2*A* ^i^	0.94	2.97	3.51 (1)	117
C205—H205⋯*Cg* ^ii^	0.94	2.86	3.749 (9)	159

Symmetry codes: (i) 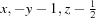; (ii) 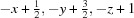.

**Table 4:** Experimental details

	**3**	**4**
Crystal data		
Chemical formula	[IrCl_2_(C_58_H_51_N_3_P_4_)]Cl·5.5C_2_H_3_N	[IrI(C_26_H_22_N_2_P_2_)(C_26_H_22_P_2_)(C_6_H_7_N](I)(I_3_)·0.5I_2_·CH_4_O·0.5CH_2_Cl_2_
*M*_r_	1438.24	1942.00
Crystal system, space group	Monoclinic, *P*2_1_/*n*	Monoclinic, *C*2/*c*
Temperature (K)	293	233
*a*, *b*, *c* (Å)	15.8874 (2), 21.0665 (3), 23.2646 (3)	37.2962 (3), 18.7310 (2), 19.2348 (2)
β (°)	106.107 (1)	106.631 (1)
*V* (Å^3^)	7480.82 (18)	12875.2 (2)
*Z*	4	8
Radiation type	Mo *K*α	Mo *K*α
μ (mm^−1^)	2.02	5.13
Crystal size (mm)	0.31 × 0.08 × 0.04	0.32 × 0.19 × 0.14

Data collection		
Diffractometer	Nonius KappaCCD	Nonius KappaCCD
No. of measured, independent and observed [*I* > 2σ(*I*)] reflections	47352, 13143, 10797	40122, 11285, 10348
*R*_int_	0.049	0.035
(sin θ/λ)_max_ (Å^−1^)	0.595	0.595

Refinement		
*R*[*F* ^2^ > 2σ(*F* ^2^)], *wR*(*F* ^2^), *S*	0.039, 0.100, 1.08	0.041, 0.114, 1.11
No. of reflections	13143	11285
No. of parameters	734	732
No. of restraints	27	3
H-atom treatment	H atoms treated by a mixture of independent and constrained refinement	H atoms treated by a mixture of independent and constrained refinement
Δρ_max_, Δρ_min_ (e Å^−3^)	0.77, −0.44	1.30, −2.92

Computer programs: *COLLECT* (Nonius, 1998 ▸), *DENZO* and *SCALEPACK* (Otwinowski & Minor, 1997 ▸), *SHELXS97* (Sheldrick, 2008 ▸), *SHELXL2014* (Sheldrick, 2015 ▸), *Mercury* (Macrae *et al.*, 2008 ▸) and *publCIF* (Westrip, 2010 ▸).

## supplementary crystallographic information

### Dichlorido(phenylmethanimine-κN)(1,1,3,3,7,7,9,9-octaphenyl-4,5-diaza-1,3λ5,7λ4,9-tetraphosphanona-3,5-dien-6-yl-κ2P1,P9)iridium(III) chloride acetonitrile hemihendecasolvate (3) Crystal data

**Table:**

[IrCl_2_(C_58_H_51_N_3_P_4_)]Cl·5.5C_2_H_3_N	*F*(000) = 2916
*M**_r_* = 1438.24	*D*_x_ = 1.277 Mg m^−^^3^
Monoclinic, *P*2_1_/*n*	Mo *K*α radiation, λ = 0.71073 Å
*a* = 15.8874 (2) Å	Cell parameters from 117075 reflections
*b* = 21.0665 (3) Å	θ = 1.0–26.0°
*c* = 23.2646 (3) Å	µ = 2.02 mm^−^^1^
β = 106.107 (1)°	*T* = 293 K
*V* = 7480.82 (18) Å^3^	Prism, colorless
*Z* = 4	0.31 × 0.08 × 0.04 mm

### Dichlorido(phenylmethanimine-κN)(1,1,3,3,7,7,9,9-octaphenyl-4,5-diaza-1,3λ5,7λ4,9-tetraphosphanona-3,5-dien-6-yl-κ2P1,P9)iridium(III) chloride acetonitrile hemihendecasolvate (3) Data collection

**Table:**

Nonius KappaCCD diffractometer	*R*_int_ = 0.049
phi– and ω–scans	θ_max_ = 25.0°, θ_min_ = 1.7°
47352 measured reflections	*h* = −18→18
13143 independent reflections	*k* = −25→25
10797 reflections with *I* > 2σ(*I*)	*l* = −27→27

### Dichlorido(phenylmethanimine-κN)(1,1,3,3,7,7,9,9-octaphenyl-4,5-diaza-1,3λ5,7λ4,9-tetraphosphanona-3,5-dien-6-yl-κ2P1,P9)iridium(III) chloride acetonitrile hemihendecasolvate (3) Refinement

**Table:**

Refinement on *F*^2^	27 restraints
Least-squares matrix: full	Hydrogen site location: mixed
*R*[*F*^2^ > 2σ(*F*^2^)] = 0.039	H atoms treated by a mixture of independent and constrained refinement
*wR*(*F*^2^) = 0.100	*w* = 1/[σ^2^(*F*_o_^2^) + (0.0372*P*)^2^ + 16.2457*P*] where *P* = (*F*_o_^2^ + 2*F*_c_^2^)/3
*S* = 1.08	(Δ/σ)_max_ = 0.001
13143 reflections	Δρ_max_ = 0.77 e Å^−^^3^
734 parameters	Δρ_min_ = −0.44 e Å^−^^3^

### Dichlorido(phenylmethanimine-κN)(1,1,3,3,7,7,9,9-octaphenyl-4,5-diaza-1,3λ5,7λ4,9-tetraphosphanona-3,5-dien-6-yl-κ2P1,P9)iridium(III) chloride acetonitrile hemihendecasolvate (3) Special details

**Table:**

Experimental. All data sets were measured with several scans to increase the number of redundant reflections. In our experience this method of averaging redundant reflections replaces in a good approximation semi-empirical absorptions methods (absorption correction programs like SORTAV lead to no better data sets).
Geometry. All esds (except the esd in the dihedral angle between two l.s. planes) are estimated using the full covariance matrix. The cell esds are taken into account individually in the estimation of esds in distances, angles and torsion angles; correlations between esds in cell parameters are only used when they are defined by crystal symmetry. An approximate (isotropic) treatment of cell esds is used for estimating esds involving l.s. planes.
Refinement. The hydrogen at N3 was found and refined normally with isotropic displacement parameters. The solvent molecules of acetonitrile in the crystal lattice are strongly disordered in position and occupation. At least a sum of 5.5 molecules in the asymmetric units were refined. Occupation values were varied to a more or less reasonable isotropic displacement factor. All C and N-atoms of solvents were isotropically refined with a sum of bond restraints and hydrogen atoms were omitted.

### Dichlorido(phenylmethanimine-κN)(1,1,3,3,7,7,9,9-octaphenyl-4,5-diaza-1,3λ5,7λ4,9-tetraphosphanona-3,5-dien-6-yl-κ2P1,P9)iridium(III) chloride acetonitrile hemihendecasolvate (3) Fractional atomic coordinates and isotropic or equivalent isotropic displacement parameters (Å^2^)

**Table:**

	*x*	*y*	*z*	*U*_iso_*/*U*_eq_	Occ. (<1)
Ir1	0.85062 (2)	0.50587 (2)	0.13892 (2)	0.03393 (7)	
P1	0.87793 (8)	0.57823 (5)	0.21646 (5)	0.0379 (3)	
P2	0.72156 (8)	0.63106 (5)	0.12389 (6)	0.0394 (3)	
P3	0.61365 (7)	0.41793 (5)	0.11211 (5)	0.0356 (3)	
P4	0.80771 (7)	0.42085 (5)	0.18720 (5)	0.0347 (3)	
Cl1	0.99975 (8)	0.46384 (6)	0.15473 (6)	0.0479 (3)	
Cl2	0.89854 (8)	0.58983 (5)	0.08509 (6)	0.0481 (3)	
Cl3	0.63193 (10)	0.30927 (6)	0.28422 (6)	0.0627 (4)	
N1	0.6530 (2)	0.52482 (16)	0.08207 (16)	0.0376 (9)	
N2	0.6460 (2)	0.45866 (15)	0.06499 (16)	0.0361 (8)	
N3	0.8199 (3)	0.45600 (17)	0.05876 (17)	0.0375 (9)	
H3N	0.771 (3)	0.440 (2)	0.0499 (19)	0.034 (13)*	
C1	0.7285 (3)	0.54535 (19)	0.11152 (19)	0.0372 (10)	
C2	0.8197 (3)	0.6504 (2)	0.1823 (2)	0.0416 (11)	
H2A	0.8041	0.6754	0.2129	0.050*	
H2B	0.8581	0.6759	0.1657	0.050*	
C3	0.6916 (3)	0.4194 (2)	0.18611 (19)	0.0368 (10)	
H3A	0.6817	0.3822	0.2080	0.044*	
H3B	0.6797	0.4565	0.2073	0.044*	
C4	0.8626 (3)	0.4518 (2)	0.0201 (2)	0.0449 (11)	
H4	0.9153	0.4738	0.0281	0.054*	
C5	0.8365 (3)	0.4158 (2)	−0.0357 (2)	0.0469 (12)	
C6	0.7747 (3)	0.3677 (3)	−0.0448 (3)	0.0602 (14)	
H6	0.7489	0.3572	−0.0147	0.072*	
C7	0.7516 (4)	0.3353 (3)	−0.0985 (3)	0.0771 (19)	
H7	0.7104	0.3028	−0.1048	0.092*	
C8	0.7903 (5)	0.3517 (4)	−0.1427 (3)	0.088 (2)	
H8	0.7738	0.3305	−0.1792	0.106*	
C9	0.8513 (5)	0.3977 (4)	−0.1343 (3)	0.0802 (19)	
H9	0.8771	0.4080	−0.1645	0.096*	
C10	0.8752 (4)	0.4296 (3)	−0.0800 (2)	0.0596 (14)	
H10	0.9182	0.4608	−0.0736	0.072*	
C101	0.9882 (3)	0.6079 (2)	0.2502 (2)	0.0432 (11)	
C102	1.0578 (3)	0.5652 (3)	0.2687 (2)	0.0606 (15)	
H102	1.0482	0.5219	0.2629	0.073*	
C103	1.1406 (4)	0.5878 (3)	0.2956 (3)	0.0753 (19)	
H103	1.1867	0.5592	0.3083	0.090*	
C104	1.1566 (4)	0.6511 (3)	0.3040 (3)	0.0738 (18)	
H104	1.2130	0.6657	0.3220	0.089*	
C105	1.0047 (4)	0.6722 (3)	0.2596 (3)	0.0601 (15)	
H105	0.9590	0.7013	0.2484	0.072*	
C106	1.0888 (4)	0.6930 (3)	0.2857 (3)	0.0776 (19)	
H106	1.0997	0.7363	0.2909	0.093*	
C107	0.8336 (3)	0.5665 (2)	0.2801 (2)	0.0452 (12)	
C108	0.7437 (4)	0.5627 (2)	0.2698 (3)	0.0522 (13)	
H108	0.7078	0.5646	0.2307	0.063*	
C109	0.7058 (4)	0.5560 (3)	0.3172 (3)	0.0666 (16)	
H109	0.6453	0.5530	0.3098	0.080*	
C110	0.7584 (5)	0.5538 (3)	0.3740 (3)	0.0745 (18)	
H110	0.7337	0.5496	0.4057	0.089*	
C111	0.8474 (5)	0.5578 (3)	0.3852 (3)	0.0743 (18)	
H111	0.8824	0.5564	0.4246	0.089*	
C112	0.8872 (4)	0.5639 (2)	0.3384 (2)	0.0568 (14)	
H112	0.9478	0.5663	0.3464	0.068*	
C201	0.7162 (3)	0.6777 (2)	0.0582 (2)	0.0460 (12)	
C202	0.7523 (4)	0.7378 (2)	0.0635 (3)	0.0645 (16)	
H202	0.7830	0.7536	0.1007	0.077*	
C203	0.7421 (5)	0.7739 (3)	0.0128 (3)	0.084 (2)	
H203	0.7654	0.8147	0.0159	0.101*	
C204	0.6976 (5)	0.7503 (3)	−0.0428 (3)	0.0795 (19)	
H204	0.6924	0.7746	−0.0770	0.095*	
C205	0.6614 (4)	0.6915 (3)	−0.0473 (3)	0.0731 (18)	
H205	0.6306	0.6760	−0.0846	0.088*	
C206	0.6700 (4)	0.6548 (2)	0.0026 (2)	0.0601 (15)	
H206	0.6447	0.6147	−0.0008	0.072*	
C207	0.6287 (3)	0.6555 (2)	0.1492 (2)	0.0450 (12)	
C208	0.5559 (4)	0.6182 (3)	0.1437 (3)	0.0749 (19)	
H208	0.5529	0.5778	0.1270	0.090*	
C209	0.4863 (4)	0.6415 (3)	0.1635 (4)	0.096 (3)	
H209	0.4377	0.6159	0.1611	0.116*	
C210	0.4891 (4)	0.7007 (3)	0.1861 (3)	0.083 (2)	
H210	0.4426	0.7158	0.1992	0.099*	
C211	0.5596 (4)	0.7381 (3)	0.1895 (3)	0.081 (2)	
H211	0.5605	0.7794	0.2038	0.097*	
C212	0.6299 (4)	0.7159 (2)	0.1720 (3)	0.0646 (16)	
H212	0.6786	0.7418	0.1756	0.078*	
C301	0.5921 (3)	0.3392 (2)	0.0821 (2)	0.0420 (11)	
C302	0.5830 (4)	0.3293 (2)	0.0220 (2)	0.0564 (14)	
H302	0.5922	0.3623	−0.0020	0.068*	
C303	0.5599 (5)	0.2691 (3)	−0.0021 (3)	0.084 (2)	
H303	0.5553	0.2616	−0.0423	0.101*	
C304	0.5442 (5)	0.2211 (3)	0.0328 (3)	0.084 (2)	
H304	0.5283	0.1811	0.0164	0.100*	
C305	0.5516 (4)	0.2315 (3)	0.0919 (3)	0.0765 (19)	
H305	0.5409	0.1985	0.1155	0.092*	
C306	0.5747 (4)	0.2904 (2)	0.1169 (2)	0.0570 (14)	
H306	0.5786	0.2973	0.1570	0.068*	
C307	0.5123 (3)	0.4445 (2)	0.1256 (2)	0.0407 (11)	
C308	0.4517 (3)	0.4752 (2)	0.0794 (2)	0.0470 (12)	
H308	0.4651	0.4833	0.0436	0.056*	
C309	0.3723 (4)	0.4934 (2)	0.0862 (3)	0.0592 (15)	
H309	0.3316	0.5131	0.0546	0.071*	
C310	0.3522 (4)	0.4831 (3)	0.1387 (3)	0.0737 (18)	
H310	0.2985	0.4965	0.1431	0.088*	
C311	0.4114 (4)	0.4529 (4)	0.1852 (3)	0.082 (2)	
H311	0.3976	0.4457	0.2210	0.099*	
C312	0.4917 (4)	0.4331 (3)	0.1787 (3)	0.0619 (15)	
H312	0.5315	0.4123	0.2100	0.074*	
C401	0.8239 (3)	0.3406 (2)	0.1607 (2)	0.0422 (11)	
C402	0.8774 (4)	0.3276 (2)	0.1248 (2)	0.0549 (13)	
H402	0.9069	0.3607	0.1122	0.066*	
C403	0.8882 (4)	0.2658 (3)	0.1069 (3)	0.0736 (18)	
H403	0.9241	0.2576	0.0823	0.088*	
C404	0.8451 (5)	0.2167 (3)	0.1260 (3)	0.0783 (19)	
H404	0.8509	0.1754	0.1136	0.094*	
C405	0.7938 (4)	0.2291 (2)	0.1634 (3)	0.0701 (17)	
H405	0.7659	0.1959	0.1771	0.084*	
C406	0.7832 (4)	0.2902 (2)	0.1807 (3)	0.0546 (13)	
H406	0.7485	0.2980	0.2062	0.065*	
C407	0.8643 (3)	0.4134 (2)	0.2671 (2)	0.0427 (11)	
C408	0.8210 (4)	0.4055 (2)	0.3110 (2)	0.0503 (12)	
H408	0.7601	0.4056	0.3007	0.060*	
C409	0.8686 (4)	0.3974 (3)	0.3701 (2)	0.0626 (15)	
H409	0.8394	0.3918	0.3993	0.075*	
C410	0.9592 (4)	0.3976 (3)	0.3860 (3)	0.0713 (18)	
H410	0.9906	0.3925	0.4259	0.086*	
C411	1.0026 (4)	0.4053 (3)	0.3439 (3)	0.0750 (18)	
H411	1.0635	0.4058	0.3549	0.090*	
C412	0.9556 (3)	0.4125 (3)	0.2837 (2)	0.0567 (14)	
H412	0.9854	0.4167	0.2547	0.068*	
N4	0.9986 (11)	0.7744 (9)	0.0154 (7)	0.242 (8)*	0.8
C11	1.0108 (11)	0.7622 (8)	0.0658 (7)	0.170 (6)*	0.8
C12	1.0126 (7)	0.7481 (5)	0.1245 (5)	0.114 (3)*	0.8
N5	0.8063 (14)	0.6958 (9)	−0.3079 (9)	0.240 (8)*	0.7
C13	0.7960 (14)	0.6511 (9)	−0.2805 (9)	0.193 (8)*	0.7
C14	0.7862 (10)	0.5920 (7)	−0.2552 (7)	0.132 (5)*	0.7
N6	0.1837 (13)	0.3904 (10)	−0.5292 (9)	0.245 (9)*	0.7
C15	0.2378 (13)	0.3567 (9)	−0.5377 (8)	0.163 (6)*	0.7
C16	0.3195 (16)	0.3349 (13)	−0.5382 (12)	0.257 (12)*	0.7
N7	0.0715 (11)	0.5571 (8)	−0.5313 (7)	0.212 (6)*	0.8
C17	0.1364 (11)	0.5537 (8)	−0.5404 (7)	0.154 (5)*	0.8
C18	0.2271 (12)	0.5480 (10)	−0.5379 (9)	0.220 (8)*	0.8
N8	0.8726 (13)	0.6302 (9)	−0.0689 (8)	0.125 (6)*	0.5
C19	0.823 (3)	0.654 (2)	−0.1087 (19)	0.34 (3)*	0.5
C20	0.776 (2)	0.6924 (16)	−0.1596 (12)	0.215 (13)*	0.5
N8A	0.8369 (14)	0.7278 (9)	−0.1420 (9)	0.159 (7)*	0.5
C19A	0.8575 (13)	0.6878 (10)	−0.1068 (8)	0.115 (6)*	0.5
C20A	0.9118 (15)	0.6550 (11)	−0.0539 (9)	0.134 (8)*	0.5
N9	0.3776 (13)	0.5841 (10)	−0.3990 (9)	0.172 (7)*	0.5
C21	0.4509 (15)	0.5722 (11)	−0.3647 (10)	0.147 (8)*	0.5
C22	0.5442 (15)	0.5790 (17)	−0.3576 (15)	0.236 (14)*	0.5
N9A	0.4319 (17)	0.4368 (12)	−0.4302 (12)	0.202 (9)*	0.5
C21A	0.485 (2)	0.4212 (17)	−0.3857 (14)	0.236 (15)*	0.5
C22A	0.566 (3)	0.381 (2)	−0.3607 (19)	0.32 (2)*	0.5
N10	0.6366 (18)	0.4471 (13)	−0.2927 (12)	0.228 (11)*	0.5
C23	0.6014 (19)	0.3984 (14)	−0.3021 (14)	0.177 (10)*	0.5
C24	0.569 (2)	0.3356 (15)	−0.3187 (17)	0.238 (15)*	0.5

### Dichlorido(phenylmethanimine-κN)(1,1,3,3,7,7,9,9-octaphenyl-4,5-diaza-1,3λ5,7λ4,9-tetraphosphanona-3,5-dien-6-yl-κ2P1,P9)iridium(III) chloride acetonitrile hemihendecasolvate (3) Atomic displacement parameters (Å^2^)

**Table:**

	*U*^11^	*U*^22^	*U*^33^	*U*^12^	*U*^13^	*U*^23^
Ir1	0.03548 (11)	0.02598 (10)	0.03721 (11)	−0.00083 (7)	0.00487 (7)	0.00162 (7)
P1	0.0399 (7)	0.0288 (6)	0.0402 (6)	−0.0016 (5)	0.0033 (5)	0.0001 (5)
P2	0.0404 (7)	0.0232 (5)	0.0480 (7)	−0.0019 (5)	0.0012 (5)	0.0011 (5)
P3	0.0384 (6)	0.0243 (5)	0.0412 (6)	−0.0022 (4)	0.0063 (5)	0.0004 (5)
P4	0.0370 (6)	0.0256 (5)	0.0383 (6)	0.0031 (4)	0.0050 (5)	0.0024 (5)
Cl1	0.0395 (6)	0.0454 (7)	0.0552 (7)	0.0034 (5)	0.0070 (5)	0.0033 (5)
Cl2	0.0501 (7)	0.0378 (6)	0.0566 (7)	−0.0046 (5)	0.0150 (6)	0.0070 (5)
Cl3	0.0773 (9)	0.0441 (7)	0.0646 (9)	−0.0076 (6)	0.0165 (7)	0.0147 (6)
N1	0.044 (2)	0.0241 (18)	0.041 (2)	−0.0015 (16)	0.0062 (18)	−0.0002 (16)
N2	0.039 (2)	0.0242 (18)	0.042 (2)	−0.0032 (15)	0.0064 (17)	−0.0026 (15)
N3	0.036 (2)	0.034 (2)	0.042 (2)	−0.0035 (17)	0.0104 (19)	−0.0025 (17)
C1	0.044 (3)	0.026 (2)	0.038 (2)	0.0010 (19)	0.005 (2)	0.0040 (18)
C2	0.043 (3)	0.027 (2)	0.049 (3)	−0.0036 (19)	0.002 (2)	−0.001 (2)
C3	0.042 (3)	0.026 (2)	0.040 (2)	0.0016 (18)	0.007 (2)	0.0028 (18)
C4	0.042 (3)	0.040 (3)	0.051 (3)	−0.004 (2)	0.010 (2)	0.000 (2)
C5	0.041 (3)	0.048 (3)	0.050 (3)	0.004 (2)	0.011 (2)	−0.007 (2)
C6	0.052 (3)	0.066 (4)	0.064 (4)	−0.008 (3)	0.019 (3)	−0.016 (3)
C7	0.061 (4)	0.083 (4)	0.087 (5)	−0.015 (3)	0.021 (4)	−0.035 (4)
C8	0.074 (5)	0.115 (6)	0.068 (4)	0.009 (4)	0.007 (4)	−0.041 (4)
C9	0.080 (5)	0.109 (6)	0.059 (4)	−0.008 (4)	0.031 (3)	−0.021 (4)
C10	0.053 (3)	0.071 (4)	0.058 (3)	0.000 (3)	0.021 (3)	−0.009 (3)
C101	0.042 (3)	0.043 (3)	0.038 (3)	−0.004 (2)	0.000 (2)	−0.004 (2)
C102	0.050 (3)	0.059 (3)	0.061 (3)	0.003 (3)	−0.005 (3)	−0.013 (3)
C103	0.052 (4)	0.089 (5)	0.073 (4)	0.011 (3)	−0.003 (3)	−0.024 (4)
C104	0.045 (3)	0.099 (5)	0.068 (4)	−0.014 (3)	0.001 (3)	−0.021 (4)
C105	0.051 (3)	0.047 (3)	0.070 (4)	−0.008 (2)	−0.004 (3)	−0.006 (3)
C106	0.072 (4)	0.064 (4)	0.081 (4)	−0.024 (3)	−0.005 (3)	−0.014 (3)
C107	0.066 (3)	0.025 (2)	0.045 (3)	−0.003 (2)	0.015 (2)	−0.0015 (19)
C108	0.061 (3)	0.031 (3)	0.066 (3)	0.000 (2)	0.020 (3)	0.001 (2)
C109	0.081 (4)	0.043 (3)	0.089 (5)	0.004 (3)	0.046 (4)	0.002 (3)
C110	0.105 (6)	0.052 (4)	0.081 (5)	−0.008 (3)	0.050 (4)	−0.008 (3)
C111	0.118 (6)	0.055 (4)	0.052 (3)	−0.009 (4)	0.026 (4)	−0.003 (3)
C112	0.075 (4)	0.042 (3)	0.052 (3)	−0.004 (3)	0.017 (3)	0.000 (2)
C201	0.045 (3)	0.032 (2)	0.052 (3)	−0.001 (2)	0.000 (2)	0.006 (2)
C202	0.079 (4)	0.039 (3)	0.065 (4)	−0.015 (3)	0.001 (3)	0.012 (3)
C203	0.104 (5)	0.045 (3)	0.091 (5)	−0.020 (3)	0.009 (4)	0.023 (3)
C204	0.095 (5)	0.069 (4)	0.062 (4)	−0.004 (4)	0.000 (4)	0.025 (3)
C205	0.082 (4)	0.069 (4)	0.054 (4)	−0.011 (3)	−0.003 (3)	0.014 (3)
C206	0.071 (4)	0.042 (3)	0.055 (3)	−0.007 (3)	−0.004 (3)	0.006 (2)
C207	0.042 (3)	0.030 (2)	0.059 (3)	0.004 (2)	0.006 (2)	−0.001 (2)
C208	0.053 (4)	0.048 (3)	0.122 (6)	−0.005 (3)	0.021 (4)	−0.030 (3)
C209	0.056 (4)	0.071 (4)	0.173 (8)	−0.013 (3)	0.049 (5)	−0.046 (5)
C210	0.058 (4)	0.063 (4)	0.127 (6)	0.007 (3)	0.024 (4)	−0.036 (4)
C211	0.072 (4)	0.049 (3)	0.112 (5)	0.005 (3)	0.012 (4)	−0.032 (3)
C212	0.057 (3)	0.039 (3)	0.096 (5)	−0.003 (2)	0.018 (3)	−0.020 (3)
C301	0.044 (3)	0.032 (2)	0.047 (3)	−0.003 (2)	0.006 (2)	−0.005 (2)
C302	0.068 (4)	0.041 (3)	0.060 (3)	−0.013 (3)	0.017 (3)	−0.010 (2)
C303	0.120 (6)	0.061 (4)	0.075 (4)	−0.026 (4)	0.033 (4)	−0.032 (3)
C304	0.114 (6)	0.042 (3)	0.094 (5)	−0.026 (3)	0.027 (4)	−0.026 (3)
C305	0.100 (5)	0.035 (3)	0.083 (5)	−0.015 (3)	0.007 (4)	0.002 (3)
C306	0.074 (4)	0.033 (3)	0.057 (3)	−0.010 (2)	0.006 (3)	0.001 (2)
C307	0.037 (3)	0.031 (2)	0.051 (3)	−0.0031 (19)	0.009 (2)	−0.001 (2)
C308	0.043 (3)	0.041 (3)	0.052 (3)	0.002 (2)	0.005 (2)	−0.001 (2)
C309	0.045 (3)	0.054 (3)	0.070 (4)	0.009 (2)	0.002 (3)	0.002 (3)
C310	0.045 (3)	0.086 (4)	0.092 (5)	0.010 (3)	0.022 (3)	−0.004 (4)
C311	0.064 (4)	0.112 (6)	0.078 (4)	0.013 (4)	0.031 (4)	0.015 (4)
C312	0.050 (3)	0.078 (4)	0.059 (3)	0.011 (3)	0.017 (3)	0.015 (3)
C401	0.046 (3)	0.032 (2)	0.045 (3)	0.008 (2)	0.006 (2)	0.002 (2)
C402	0.066 (3)	0.032 (3)	0.071 (4)	0.004 (2)	0.027 (3)	0.003 (2)
C403	0.092 (5)	0.047 (3)	0.097 (5)	0.013 (3)	0.052 (4)	−0.004 (3)
C404	0.109 (5)	0.027 (3)	0.109 (5)	0.006 (3)	0.046 (4)	−0.002 (3)
C405	0.092 (5)	0.031 (3)	0.100 (5)	0.002 (3)	0.047 (4)	0.005 (3)
C406	0.066 (3)	0.028 (2)	0.076 (4)	0.003 (2)	0.030 (3)	0.007 (2)
C407	0.054 (3)	0.026 (2)	0.040 (3)	0.003 (2)	0.001 (2)	0.0065 (19)
C408	0.054 (3)	0.042 (3)	0.050 (3)	−0.002 (2)	0.007 (3)	0.003 (2)
C409	0.085 (4)	0.055 (3)	0.045 (3)	−0.003 (3)	0.014 (3)	0.012 (3)
C410	0.085 (5)	0.077 (4)	0.038 (3)	0.000 (3)	−0.004 (3)	0.016 (3)
C411	0.060 (4)	0.085 (4)	0.062 (4)	0.001 (3)	−0.015 (3)	0.017 (3)
C412	0.053 (3)	0.059 (3)	0.052 (3)	0.003 (3)	0.004 (3)	0.014 (3)

### Dichlorido(phenylmethanimine-κN)(1,1,3,3,7,7,9,9-octaphenyl-4,5-diaza-1,3λ5,7λ4,9-tetraphosphanona-3,5-dien-6-yl-κ2P1,P9)iridium(III) chloride acetonitrile hemihendecasolvate (3) Geometric parameters (Å, º)

**Table:**

Ir1—C1	2.044 (4)	C210—C211	1.355 (9)
Ir1—N3	2.077 (4)	C211—C212	1.372 (8)
Ir1—P1	2.3090 (12)	C301—C302	1.381 (7)
Ir1—P4	2.3151 (11)	C301—C306	1.384 (7)
Ir1—Cl2	2.4094 (11)	C302—C303	1.395 (7)
Ir1—Cl1	2.4595 (12)	C303—C304	1.364 (9)
P1—C101	1.821 (5)	C304—C305	1.365 (9)
P1—C107	1.825 (5)	C305—C306	1.375 (7)
P1—C2	1.842 (4)	C307—C312	1.383 (7)
P2—C201	1.800 (5)	C307—C308	1.389 (6)
P2—C2	1.808 (4)	C308—C309	1.370 (7)
P2—C207	1.808 (5)	C309—C310	1.363 (8)
P2—C1	1.837 (4)	C310—C311	1.378 (9)
P3—N2	1.586 (4)	C311—C312	1.389 (8)
P3—C301	1.795 (4)	C401—C402	1.375 (7)
P3—C307	1.812 (5)	C401—C406	1.389 (7)
P3—C3	1.821 (4)	C402—C403	1.392 (7)
P4—C407	1.832 (5)	C403—C404	1.379 (8)
P4—C3	1.837 (5)	C404—C405	1.373 (8)
P4—C401	1.842 (5)	C405—C406	1.374 (7)
N1—C1	1.280 (5)	C407—C408	1.392 (7)
N1—N2	1.445 (5)	C407—C412	1.394 (7)
N3—H3N	0.81 (5)	C408—C409	1.382 (7)
N3—C4	1.270 (6)	C409—C410	1.384 (8)
C4—C5	1.462 (7)	C410—C411	1.355 (9)
C5—C10	1.371 (7)	C411—C412	1.400 (7)
C5—C6	1.386 (7)	N4—C11	1.162 (13)
C6—C7	1.380 (8)	C11—C12	1.391 (13)
C7—C8	1.382 (10)	N5—C13	1.175 (14)
C8—C9	1.347 (10)	C13—C14	1.403 (14)
C9—C10	1.387 (8)	N6—C15	1.172 (14)
C101—C105	1.385 (7)	C15—C16	1.380 (15)
C101—C102	1.397 (7)	N7—C17	1.112 (13)
C102—C103	1.375 (8)	C17—C18	1.432 (13)
C103—C104	1.362 (9)	N8—C20A	0.81 (3)
C104—C106	1.365 (9)	N8—C19	1.152 (17)
C105—C106	1.377 (8)	N8—C19A	1.48 (3)
C107—C108	1.382 (7)	C19—C19A	0.88 (6)
C107—C112	1.388 (7)	C19—C20	1.456 (17)
C108—C109	1.404 (8)	C19—C20A	1.61 (4)
C109—C110	1.354 (9)	C19—N8A	1.77 (5)
C110—C111	1.368 (9)	C20—N8A	1.20 (4)
C111—C112	1.409 (8)	C20—C19A	1.52 (3)
C201—C202	1.382 (7)	N8A—C19A	1.158 (14)
C201—C206	1.383 (7)	C19A—C20A	1.465 (15)
C202—C203	1.373 (8)	N9—C21	1.242 (15)
C203—C204	1.385 (9)	C21—C22	1.453 (16)
C204—C205	1.356 (8)	N9A—C21A	1.183 (17)
C205—C206	1.371 (7)	C21A—C22A	1.510 (18)
C207—C212	1.376 (7)	C22A—C24	1.36 (5)
C207—C208	1.376 (7)	C22A—C23	1.37 (5)
C208—C209	1.399 (8)	N10—C23	1.159 (15)
C209—C210	1.350 (8)	C23—C24	1.433 (16)
			
C1—Ir1—N3	87.68 (17)	C208—C207—P2	123.3 (4)
C1—Ir1—P1	86.06 (12)	C207—C208—C209	119.4 (5)
N3—Ir1—P1	169.02 (11)	C210—C209—C208	120.6 (6)
C1—Ir1—P4	94.83 (12)	C209—C210—C211	119.9 (6)
N3—Ir1—P4	90.96 (11)	C210—C211—C212	120.8 (5)
P1—Ir1—P4	98.54 (4)	C211—C212—C207	120.3 (5)
C1—Ir1—Cl2	87.06 (12)	C302—C301—C306	119.8 (4)
N3—Ir1—Cl2	86.22 (11)	C302—C301—P3	119.3 (4)
P1—Ir1—Cl2	84.45 (4)	C306—C301—P3	120.5 (4)
P4—Ir1—Cl2	176.55 (4)	C301—C302—C303	119.2 (5)
C1—Ir1—Cl1	170.06 (13)	C304—C303—C302	120.3 (6)
N3—Ir1—Cl1	85.93 (12)	C303—C304—C305	120.2 (5)
P1—Ir1—Cl1	98.99 (4)	C304—C305—C306	120.6 (6)
P4—Ir1—Cl1	92.87 (4)	C305—C306—C301	119.8 (5)
Cl2—Ir1—Cl1	84.94 (4)	C312—C307—C308	119.1 (5)
C101—P1—C107	103.8 (2)	C312—C307—P3	122.9 (4)
C101—P1—C2	102.2 (2)	C308—C307—P3	117.9 (4)
C107—P1—C2	101.6 (2)	C309—C308—C307	120.3 (5)
C101—P1—Ir1	120.89 (16)	C310—C309—C308	120.7 (5)
C107—P1—Ir1	120.87 (15)	C309—C310—C311	119.9 (6)
C2—P1—Ir1	104.20 (15)	C310—C311—C312	120.1 (6)
C201—P2—C2	110.1 (2)	C307—C312—C311	119.8 (5)
C201—P2—C207	105.7 (2)	C402—C401—C406	118.5 (4)
C2—P2—C207	107.6 (2)	C402—C401—P4	123.6 (4)
C201—P2—C1	113.2 (2)	C406—C401—P4	117.9 (4)
C2—P2—C1	105.3 (2)	C401—C402—C403	121.0 (5)
C207—P2—C1	114.9 (2)	C404—C403—C402	119.5 (6)
N2—P3—C301	107.1 (2)	C405—C404—C403	119.8 (5)
N2—P3—C307	115.3 (2)	C404—C405—C406	120.4 (5)
C301—P3—C307	105.6 (2)	C405—C406—C401	120.8 (5)
N2—P3—C3	112.2 (2)	C408—C407—C412	118.8 (4)
C301—P3—C3	112.9 (2)	C408—C407—P4	123.5 (4)
C307—P3—C3	103.6 (2)	C412—C407—P4	117.6 (4)
C407—P4—C3	102.8 (2)	C409—C408—C407	120.0 (5)
C407—P4—C401	100.7 (2)	C408—C409—C410	120.5 (6)
C3—P4—C401	102.3 (2)	C411—C410—C409	120.4 (5)
C407—P4—Ir1	115.50 (15)	C410—C411—C412	120.0 (6)
C3—P4—Ir1	115.85 (14)	C407—C412—C411	120.3 (6)
C401—P4—Ir1	117.40 (16)	N4—C11—C12	172 (2)
C1—N1—N2	117.5 (3)	N5—C13—C14	171 (3)
N1—N2—P3	110.3 (3)	N6—C15—C16	160 (3)
H3N—N3—C4	117 (3)	N7—C17—C18	167 (2)
H3N—N3—Ir1	114 (3)	C20A—N8—C19	109 (4)
C4—N3—Ir1	129.2 (3)	C20A—N8—C19A	73 (2)
N1—C1—P2	109.1 (3)	C19—N8—C19A	37 (3)
N1—C1—Ir1	134.2 (3)	C19A—C19—N8	92 (4)
P2—C1—Ir1	116.4 (2)	C19A—C19—C20	77 (3)
P2—C2—P1	111.3 (2)	N8—C19—C20	169 (6)
P3—C3—P4	115.5 (2)	C19A—C19—C20A	64 (3)
N3—C4—C5	126.1 (4)	N8—C19—C20A	28.5 (18)
C10—C5—C6	119.2 (5)	C20—C19—C20A	141 (5)
C10—C5—C4	118.9 (5)	C19A—C19—N8A	34.6 (18)
C6—C5—C4	122.0 (5)	N8—C19—N8A	127 (5)
C7—C6—C5	120.0 (6)	C20—C19—N8A	42.4 (19)
C6—C7—C8	119.2 (6)	C20A—C19—N8A	99 (3)
C9—C8—C7	121.6 (6)	N8A—C20—C19	83 (3)
C8—C9—C10	119.0 (6)	N8A—C20—C19A	48.5 (13)
C5—C10—C9	121.0 (6)	C19—C20—C19A	34 (2)
C105—C101—C102	118.8 (5)	C19A—N8A—C20	80 (2)
C105—C101—P1	121.4 (4)	C19A—N8A—C19	25.6 (17)
C102—C101—P1	119.8 (4)	C20—N8A—C19	54.7 (16)
C103—C102—C101	119.6 (5)	C19—C19A—N8A	120 (3)
C104—C103—C102	121.3 (6)	C19—C19A—C20A	83 (2)
C103—C104—C106	119.3 (6)	N8A—C19A—C20A	157 (3)
C106—C105—C101	119.9 (5)	C19—C19A—N8	51.1 (19)
C104—C106—C105	121.1 (6)	N8A—C19A—N8	170 (2)
C108—C107—C112	119.5 (5)	C20A—C19A—N8	32.1 (11)
C108—C107—P1	118.6 (4)	C19—C19A—C20	69 (2)
C112—C107—P1	121.8 (4)	N8A—C19A—C20	51.1 (16)
C107—C108—C109	121.1 (5)	C20A—C19A—C20	152 (2)
C110—C109—C108	119.2 (6)	N8—C19A—C20	120 (2)
C109—C110—C111	120.6 (6)	N8—C20A—C19A	75 (2)
C110—C111—C112	121.3 (6)	N8—C20A—C19	42 (2)
C107—C112—C111	118.3 (6)	C19A—C20A—C19	32.8 (19)
C202—C201—C206	120.2 (5)	N9—C21—C22	143 (2)
C202—C201—P2	120.3 (4)	N9A—C21A—C22A	144 (3)
C206—C201—P2	119.4 (4)	C24—C22A—C23	63 (2)
C203—C202—C201	118.9 (5)	C24—C22A—C21A	122 (4)
C202—C203—C204	120.8 (6)	C23—C22A—C21A	108 (4)
C205—C204—C203	119.7 (6)	N10—C23—C22A	118 (4)
C204—C205—C206	120.6 (6)	N10—C23—C24	170 (4)
C205—C206—C201	119.8 (5)	C22A—C23—C24	58 (2)
C212—C207—C208	119.0 (5)	C22A—C24—C23	59 (2)
C212—C207—P2	117.6 (4)		
			
C1—N1—N2—P3	96.1 (4)	C301—P3—C307—C312	88.1 (5)
C301—P3—N2—N1	172.0 (3)	C3—P3—C307—C312	−30.8 (5)
C307—P3—N2—N1	54.7 (3)	N2—P3—C307—C308	29.0 (4)
C3—P3—N2—N1	−63.5 (3)	C301—P3—C307—C308	−89.1 (4)
N2—N1—C1—P2	176.7 (3)	C3—P3—C307—C308	152.0 (4)
N2—N1—C1—Ir1	3.4 (6)	C312—C307—C308—C309	−0.5 (7)
C201—P2—C1—N1	−75.6 (4)	P3—C307—C308—C309	176.8 (4)
C2—P2—C1—N1	164.1 (3)	C307—C308—C309—C310	1.5 (8)
C207—P2—C1—N1	46.0 (4)	C308—C309—C310—C311	−1.3 (10)
C201—P2—C1—Ir1	99.0 (3)	C309—C310—C311—C312	0.3 (11)
C2—P2—C1—Ir1	−21.3 (3)	C308—C307—C312—C311	−0.5 (8)
C207—P2—C1—Ir1	−139.4 (2)	P3—C307—C312—C311	−177.7 (5)
C201—P2—C2—P1	−132.6 (3)	C310—C311—C312—C307	0.6 (10)
C207—P2—C2—P1	112.7 (3)	C407—P4—C401—C402	−110.3 (5)
C1—P2—C2—P1	−10.2 (3)	C3—P4—C401—C402	144.0 (4)
C101—P1—C2—P2	159.1 (3)	Ir1—P4—C401—C402	16.0 (5)
C107—P1—C2—P2	−93.8 (3)	C407—P4—C401—C406	66.2 (4)
Ir1—P1—C2—P2	32.5 (3)	C3—P4—C401—C406	−39.5 (4)
N2—P3—C3—P4	−35.6 (3)	Ir1—P4—C401—C406	−167.5 (3)
C301—P3—C3—P4	85.6 (3)	C406—C401—C402—C403	2.5 (8)
C307—P3—C3—P4	−160.6 (2)	P4—C401—C402—C403	178.9 (5)
C407—P4—C3—P3	−174.1 (2)	C401—C402—C403—C404	−0.6 (10)
C401—P4—C3—P3	−70.0 (3)	C402—C403—C404—C405	−1.4 (11)
Ir1—P4—C3—P3	59.0 (3)	C403—C404—C405—C406	1.6 (11)
H3N—N3—C4—C5	−7 (4)	C404—C405—C406—C401	0.3 (10)
Ir1—N3—C4—C5	−179.8 (3)	C402—C401—C406—C405	−2.3 (8)
N3—C4—C5—C10	161.9 (5)	P4—C401—C406—C405	−179.0 (5)
N3—C4—C5—C6	−18.8 (8)	C3—P4—C407—C408	2.9 (4)
C10—C5—C6—C7	−1.4 (8)	C401—P4—C407—C408	−102.4 (4)
C4—C5—C6—C7	179.4 (5)	Ir1—P4—C407—C408	130.0 (4)
C5—C6—C7—C8	−0.4 (10)	C3—P4—C407—C412	179.6 (4)
C6—C7—C8—C9	1.3 (11)	C401—P4—C407—C412	74.2 (4)
C7—C8—C9—C10	−0.5 (11)	Ir1—P4—C407—C412	−53.3 (4)
C6—C5—C10—C9	2.2 (8)	C412—C407—C408—C409	0.5 (7)
C4—C5—C10—C9	−178.5 (5)	P4—C407—C408—C409	177.2 (4)
C8—C9—C10—C5	−1.3 (10)	C407—C408—C409—C410	0.5 (8)
C107—P1—C101—C105	−88.5 (5)	C408—C409—C410—C411	−0.5 (9)
C2—P1—C101—C105	16.9 (5)	C409—C410—C411—C412	−0.6 (10)
Ir1—P1—C101—C105	131.8 (4)	C408—C407—C412—C411	−1.5 (8)
C107—P1—C101—C102	89.6 (5)	P4—C407—C412—C411	−178.4 (4)
C2—P1—C101—C102	−165.0 (4)	C410—C411—C412—C407	1.6 (9)
Ir1—P1—C101—C102	−50.1 (5)	C20A—N8—C19—C19A	8 (7)
C105—C101—C102—C103	−0.2 (8)	C20A—N8—C19—C20	28 (33)
P1—C101—C102—C103	−178.3 (5)	C19A—N8—C19—C20	20 (27)
C101—C102—C103—C104	−0.7 (10)	C19A—N8—C19—C20A	−8 (7)
C102—C103—C104—C106	0.4 (11)	C20A—N8—C19—N8A	11 (7)
C102—C101—C105—C106	1.4 (9)	C19A—N8—C19—N8A	2.5 (17)
P1—C101—C105—C106	179.5 (5)	C19A—C19—C20—N8A	0 (5)
C103—C104—C106—C105	0.9 (11)	N8—C19—C20—N8A	−21 (32)
C101—C105—C106—C104	−1.8 (10)	C20A—C19—C20—N8A	0 (8)
C101—P1—C107—C108	160.6 (4)	N8—C19—C20—C19A	−20 (28)
C2—P1—C107—C108	54.9 (4)	C20A—C19—C20—C19A	1 (4)
Ir1—P1—C107—C108	−59.6 (4)	N8A—C19—C20—C19A	0 (5)
C101—P1—C107—C112	−16.6 (4)	C19—C20—N8A—C19A	0 (3)
C2—P1—C107—C112	−122.4 (4)	C19A—C20—N8A—C19	0 (3)
Ir1—P1—C107—C112	123.2 (4)	N8—C19—N8A—C19A	−4 (3)
C112—C107—C108—C109	−0.4 (7)	C20—C19—N8A—C19A	−180 (8)
P1—C107—C108—C109	−177.7 (4)	C20A—C19—N8A—C19A	1 (4)
C107—C108—C109—C110	0.7 (8)	C19A—C19—N8A—C20	180 (8)
C108—C109—C110—C111	−0.4 (9)	N8—C19—N8A—C20	175 (8)
C109—C110—C111—C112	−0.2 (9)	C20A—C19—N8A—C20	−180 (5)
C108—C107—C112—C111	−0.2 (7)	N8—C19—C19A—N8A	176 (2)
P1—C107—C112—C111	177.0 (4)	C20—C19—C19A—N8A	0 (5)
C110—C111—C112—C107	0.5 (8)	C20A—C19—C19A—N8A	−179 (4)
C2—P2—C201—C202	−30.7 (5)	N8—C19—C19A—C20A	−4 (4)
C207—P2—C201—C202	85.1 (5)	C20—C19—C19A—C20A	180 (3)
C1—P2—C201—C202	−148.2 (4)	N8A—C19—C19A—C20A	179 (4)
C2—P2—C201—C206	154.1 (4)	C20—C19—C19A—N8	−176 (5)
C207—P2—C201—C206	−90.1 (5)	C20A—C19—C19A—N8	4 (4)
C1—P2—C201—C206	36.6 (5)	N8A—C19—C19A—N8	−176 (2)
C206—C201—C202—C203	−0.6 (9)	N8—C19—C19A—C20	176 (5)
P2—C201—C202—C203	−175.7 (5)	C20A—C19—C19A—C20	−180 (3)
C201—C202—C203—C204	−1.0 (11)	N8A—C19—C19A—C20	0 (5)
C202—C203—C204—C205	1.8 (12)	C20—N8A—C19A—C19	0 (6)
C203—C204—C205—C206	−1.2 (11)	C20—N8A—C19A—C20A	−178 (6)
C204—C205—C206—C201	−0.3 (10)	C19—N8A—C19A—C20A	−178 (11)
C202—C201—C206—C205	1.2 (9)	C19—N8A—C19A—C20	0 (6)
P2—C201—C206—C205	176.4 (5)	C20A—N8—C19A—C19	−172 (7)
C201—P2—C207—C212	−68.6 (5)	C19—N8—C19A—C20A	172 (7)
C2—P2—C207—C212	48.9 (5)	C20A—N8—C19A—C20	−176 (3)
C1—P2—C207—C212	165.8 (4)	C19—N8—C19A—C20	−4 (6)
C201—P2—C207—C208	107.7 (5)	N8A—C20—C19A—C19	180 (6)
C2—P2—C207—C208	−134.7 (5)	C19—C20—C19A—N8A	−180 (6)
C1—P2—C207—C208	−17.9 (6)	N8A—C20—C19A—C20A	179 (5)
C212—C207—C208—C209	−2.4 (10)	C19—C20—C19A—C20A	−1 (5)
P2—C207—C208—C209	−178.7 (6)	N8A—C20—C19A—N8	−177 (3)
C207—C208—C209—C210	2.1 (12)	C19—C20—C19A—N8	3 (5)
C208—C209—C210—C211	0.2 (13)	C19—N8—C20A—C19A	−5 (4)
C209—C210—C211—C212	−2.1 (12)	C19A—N8—C20A—C19	5 (4)
C210—C211—C212—C207	1.7 (11)	C19—C19A—C20A—N8	6 (5)
C208—C207—C212—C211	0.6 (9)	N8A—C19A—C20A—N8	−175 (6)
P2—C207—C212—C211	177.1 (5)	C20—C19A—C20A—N8	7 (6)
N2—P3—C301—C302	−16.6 (5)	N8A—C19A—C20A—C19	178 (9)
C307—P3—C301—C302	106.8 (4)	N8—C19A—C20A—C19	−6 (5)
C3—P3—C301—C302	−140.7 (4)	C20—C19A—C20A—C19	1 (5)
N2—P3—C301—C306	170.8 (4)	C19A—C19—C20A—N8	−171 (8)
C307—P3—C301—C306	−65.8 (5)	C20—C19—C20A—N8	−172 (10)
C3—P3—C301—C306	46.8 (5)	N8A—C19—C20A—N8	−171 (6)
C306—C301—C302—C303	−2.7 (8)	N8—C19—C20A—C19A	171 (8)
P3—C301—C302—C303	−175.3 (5)	C20—C19—C20A—C19A	−1 (4)
C301—C302—C303—C304	1.8 (10)	N8A—C19—C20A—C19A	0 (2)
C302—C303—C304—C305	−0.5 (12)	N9A—C21A—C22A—C24	−123 (7)
C303—C304—C305—C306	0.2 (11)	N9A—C21A—C22A—C23	167 (6)
C304—C305—C306—C301	−1.2 (10)	C24—C22A—C23—N10	170 (4)
C302—C301—C306—C305	2.4 (8)	C21A—C22A—C23—N10	−73 (5)
P3—C301—C306—C305	174.9 (5)	C21A—C22A—C23—C24	117 (5)
N2—P3—C307—C312	−153.8 (4)	C21A—C22A—C24—C23	−96 (5)

### Dichlorido(phenylmethanimine-κN)(1,1,3,3,7,7,9,9-octaphenyl-4,5-diaza-1,3λ5,7λ4,9-tetraphosphanona-3,5-dien-6-yl-κ2P1,P9)iridium(III) chloride acetonitrile hemihendecasolvate (3) Hydrogen-bond geometry (Å, º)

**Table:**

*D*—H···*A*	*D*—H	H···*A*	*D*···*A*	*D*—H···*A*
N3—H3*N*···N2	0.82 (5)	2.15 (5)	2.807 (6)	138 (4)
C208—H208···N1	0.93	2.41	3.088 (7)	130
C402—H402···N3	0.93	2.56	3.120 (6)	119
C102—H102···Cl1	0.93	2.71	3.329 (5)	125
C402—H402···Cl1	0.93	2.66	3.428 (5)	140
C412—H412···Cl1	0.93	2.60	3.440 (5)	151
C3—H3*A*···Cl3	0.97	2.63	3.563 (5)	162
C105—H105···Cl3^i^	0.93	2.69	3.586 (7)	162
C408—H408···Cl3	0.93	2.82	3.533 (6)	134

Symmetry code: (i) −*x*+3/2, *y*+1/2, −*z*+1/2.

### (4-Diazo-1,1,3,3,-tetraphenyl-1,3λ4-diphosphabut-4-yl-κP1)iodido[methylenebis(diphenylphosphine)-κ2P,P'](phenylmethanimine-κN)iridium(III) iodide–triiodide–dichloromethane–iodine–methanol (2/2/1/1/2) (4) Crystal data

**Table:**

[IrI(C_26_H_22_N_2_P_2_)(C_26_H_22_P_2_)(C_6_H_7_N]I·I_3_·0.5I_2_·CH_4_O·0.5CH_2_Cl_2_	*F*(000) = 7312
*M**_r_* = 1942.00	*D*_x_ = 2.004 Mg m^−^^3^
Monoclinic, *C*2/*c*	Mo *K*α radiation, λ = 0.71073 Å
*a* = 37.2962 (3) Å	Cell parameters from 93745 reflections
*b* = 18.7310 (2) Å	θ = 1.0–27.0°
*c* = 19.2348 (2) Å	µ = 5.13 mm^−^^1^
β = 106.631 (1)°	*T* = 233 K
*V* = 12875.2 (2) Å^3^	Prism, red
*Z* = 8	0.32 × 0.19 × 0.14 mm

### (4-Diazo-1,1,3,3,-tetraphenyl-1,3λ4-diphosphabut-4-yl-κP1)iodido[methylenebis(diphenylphosphine)-κ2P,P'](phenylmethanimine-κN)iridium(III) iodide–triiodide–dichloromethane–iodine–methanol (2/2/1/1/2) (4) Data collection

**Table:**

Nonius KappaCCD diffractometer	*R*_int_ = 0.035
phi– and ω–scans	θ_max_ = 25.0°, θ_min_ = 2.0°
40122 measured reflections	*h* = −44→43
11285 independent reflections	*k* = −22→22
10348 reflections with *I* > 2σ(*I*)	*l* = −22→22

### (4-Diazo-1,1,3,3,-tetraphenyl-1,3λ4-diphosphabut-4-yl-κP1)iodido[methylenebis(diphenylphosphine)-κ2P,P'](phenylmethanimine-κN)iridium(III) iodide–triiodide–dichloromethane–iodine–methanol (2/2/1/1/2) (4) Refinement

**Table:**

Refinement on *F*^2^	3 restraints
Least-squares matrix: full	Hydrogen site location: mixed
*R*[*F*^2^ > 2σ(*F*^2^)] = 0.041	H atoms treated by a mixture of independent and constrained refinement
*wR*(*F*^2^) = 0.114	*w* = 1/[σ^2^(*F*_o_^2^) + (0.049*P*)^2^ + 158.7648*P*] where *P* = (*F*_o_^2^ + 2*F*_c_^2^)/3
*S* = 1.11	(Δ/σ)_max_ = 0.087
11285 reflections	Δρ_max_ = 1.30 e Å^−^^3^
732 parameters	Δρ_min_ = −2.92 e Å^−^^3^

### (4-Diazo-1,1,3,3,-tetraphenyl-1,3λ4-diphosphabut-4-yl-κP1)iodido[methylenebis(diphenylphosphine)-κ2P,P'](phenylmethanimine-κN)iridium(III) iodide–triiodide–dichloromethane–iodine–methanol (2/2/1/1/2) (4) Special details

**Table:**

Experimental. All data sets were measured with several scans to increase the number of redundant reflections. In our experience this method of averaging redundant reflections replaces, in good approximation, semi-empirical absorption correction methods (programs such as SORTAV lead to no better data sets).
Geometry. All esds (except the esd in the dihedral angle between two l.s. planes) are estimated using the full covariance matrix. The cell esds are taken into account individually in the estimation of esds in distances, angles and torsion angles; correlations between esds in cell parameters are only used when they are defined by crystal symmetry. An approximate (isotropic) treatment of cell esds is used for estimating esds involving l.s. planes.
Refinement. Hydrogen at atom at N1 was found and refined isotropically with a bond restraint of 87 pm. The I_3_^-^ anion (I4–I6) is approx. 1:1 positionally disordered, as is the I^-^ anion with an I2:I2A ratio of 9:1. The solvent dichloromethane lies nearby a twofold rotation axis (disorder) and was refined with an occupancy of 0.5. A further disorder occurs for the solvent methanol with ratio 1:1. C- and O-atoms of methanol were refined isotropically with bond restraints of 140 pm. Hydrogen atoms at dichloromethane were omitted.

### (4-Diazo-1,1,3,3,-tetraphenyl-1,3λ4-diphosphabut-4-yl-κP1)iodido[methylenebis(diphenylphosphine)-κ2P,P'](phenylmethanimine-κN)iridium(III) iodide–triiodide–dichloromethane–iodine–methanol (2/2/1/1/2) (4) Fractional atomic coordinates and isotropic or equivalent isotropic displacement parameters (Å^2^)

**Table:**

	*x*	*y*	*z*	*U*_iso_*/*U*_eq_	Occ. (<1)
Ir1	0.11639 (2)	0.74873 (2)	0.26275 (2)	0.02540 (8)	
I1	0.04230 (2)	0.71763 (2)	0.20983 (2)	0.03876 (12)	
I3	0.24552 (2)	0.80905 (4)	0.04188 (4)	0.0793 (2)	
I2	0.23487 (3)	0.96021 (5)	0.13591 (5)	0.0582 (2)	0.9
I4	0.13471 (7)	0.81381 (13)	−0.23669 (10)	0.0861 (6)	0.5
I5	0.08532 (19)	0.7555 (3)	−0.3728 (3)	0.0512 (7)	0.5
I6	0.03254 (16)	0.7100 (3)	−0.5087 (3)	0.0634 (9)	0.5
I2A	0.2335 (2)	0.9909 (4)	0.1530 (5)	0.0579 (19)	0.1
I4A	0.14566 (8)	0.77918 (11)	−0.22728 (11)	0.0848 (6)	0.5
I5A	0.0893 (2)	0.7472 (4)	−0.3638 (4)	0.0752 (19)	0.5
I6A	0.03306 (18)	0.7104 (3)	−0.4957 (3)	0.0895 (17)	0.5
P1	0.13493 (4)	0.63702 (9)	0.22985 (9)	0.0321 (3)	
P2	0.12819 (5)	0.60579 (9)	0.37883 (9)	0.0358 (4)	
P3	0.12112 (4)	0.81498 (9)	0.16178 (8)	0.0309 (3)	
P4	0.17512 (4)	0.80175 (9)	0.28981 (8)	0.0293 (3)	
N1	0.09804 (14)	0.8404 (3)	0.3061 (3)	0.0291 (11)	
H1N	0.1117 (15)	0.877 (2)	0.305 (3)	0.026 (16)*	
N2	0.1250 (2)	0.7681 (4)	0.4709 (3)	0.0557 (18)	
N3	0.12187 (17)	0.7344 (3)	0.4229 (3)	0.0423 (14)	
C1	0.11981 (18)	0.6975 (3)	0.3644 (3)	0.0326 (13)	
C2	0.15339 (19)	0.5866 (4)	0.3141 (4)	0.0389 (15)	
H2A	0.1517	0.5354	0.3032	0.047*	
H2B	0.1799	0.5986	0.3354	0.047*	
C3	0.17213 (17)	0.8208 (4)	0.1954 (3)	0.0337 (13)	
H3A	0.1847	0.7846	0.1739	0.040*	
H3B	0.1816	0.8684	0.1888	0.040*	
C4	0.07356 (18)	0.8478 (3)	0.3397 (4)	0.0362 (14)	
H4	0.0590	0.8076	0.3430	0.043*	
C5	0.0662 (2)	0.9138 (4)	0.3734 (4)	0.0449 (17)	
C6	0.0811 (2)	0.9795 (4)	0.3619 (5)	0.062 (2)	
H6	0.0952	0.9837	0.3286	0.075*	
C7	0.0750 (3)	1.0382 (6)	0.4001 (8)	0.094 (4)	
H7	0.0852	1.0826	0.3933	0.113*	
C8	0.0542 (4)	1.0324 (8)	0.4475 (8)	0.111 (6)	
H8	0.0502	1.0730	0.4729	0.133*	
C9	0.0396 (4)	0.9702 (9)	0.4584 (6)	0.103 (5)	
H9	0.0256	0.9676	0.4919	0.123*	
C10	0.0445 (3)	0.9091 (6)	0.4213 (5)	0.068 (3)	
H10	0.0334	0.8657	0.4282	0.081*	
C101	0.17337 (19)	0.6296 (4)	0.1891 (4)	0.0389 (15)	
C102	0.1660 (2)	0.6367 (4)	0.1145 (4)	0.0505 (18)	
H102	0.1413	0.6446	0.0859	0.061*	
C103	0.1942 (3)	0.6324 (5)	0.0818 (5)	0.065 (2)	
H103	0.1887	0.6367	0.0312	0.077*	
C104	0.2310 (2)	0.6215 (5)	0.1238 (5)	0.067 (3)	
H104	0.2506	0.6201	0.1021	0.080*	
C105	0.2381 (2)	0.6129 (5)	0.1966 (5)	0.067 (2)	
H105	0.2628	0.6042	0.2247	0.080*	
C106	0.2102 (2)	0.6166 (4)	0.2304 (4)	0.0503 (18)	
H106	0.2158	0.6105	0.2809	0.060*	
C107	0.09997 (19)	0.5787 (4)	0.1706 (4)	0.0404 (15)	
C108	0.0778 (2)	0.6075 (4)	0.1047 (4)	0.0474 (17)	
H108	0.0790	0.6565	0.0951	0.057*	
C109	0.0540 (2)	0.5625 (5)	0.0535 (5)	0.063 (2)	
H109	0.0395	0.5816	0.0091	0.075*	
C110	0.0516 (2)	0.4922 (5)	0.0670 (6)	0.068 (3)	
H110	0.0357	0.4624	0.0323	0.081*	
C111	0.0726 (3)	0.4647 (5)	0.1316 (6)	0.072 (3)	
H111	0.0704	0.4159	0.1410	0.086*	
C112	0.0972 (2)	0.5069 (4)	0.1844 (5)	0.0519 (19)	
H112	0.1116	0.4867	0.2283	0.062*	
C201	0.1573 (2)	0.5867 (4)	0.4688 (4)	0.0430 (16)	
C202	0.1415 (2)	0.5952 (5)	0.5260 (4)	0.057 (2)	
H202	0.1162	0.6084	0.5166	0.068*	
C203	0.1632 (3)	0.5843 (6)	0.5964 (5)	0.071 (3)	
H203	0.1527	0.5905	0.6350	0.085*	
C204	0.2000 (3)	0.5643 (6)	0.6103 (5)	0.070 (3)	
H204	0.2147	0.5568	0.6584	0.084*	
C205	0.2151 (2)	0.5554 (6)	0.5550 (5)	0.071 (3)	
H205	0.2404	0.5417	0.5653	0.085*	
C206	0.1944 (2)	0.5660 (5)	0.4832 (5)	0.059 (2)	
H206	0.2052	0.5593	0.4452	0.071*	
C207	0.08725 (19)	0.5514 (4)	0.3634 (4)	0.0399 (15)	
C208	0.0521 (2)	0.5804 (4)	0.3541 (4)	0.0455 (17)	
H208	0.0488	0.6301	0.3537	0.055*	
C209	0.0217 (2)	0.5345 (5)	0.3453 (5)	0.056 (2)	
H209	−0.0023	0.5535	0.3386	0.068*	
C210	0.0265 (2)	0.4626 (5)	0.3464 (5)	0.063 (2)	
H210	0.0056	0.4325	0.3405	0.076*	
C211	0.0607 (3)	0.4338 (4)	0.3558 (6)	0.072 (3)	
H211	0.0635	0.3839	0.3555	0.086*	
C212	0.0920 (2)	0.4776 (4)	0.3661 (5)	0.062 (2)	
H212	0.1160	0.4575	0.3747	0.074*	
C301	0.10947 (19)	0.7897 (4)	0.0665 (3)	0.0362 (14)	
C302	0.0724 (2)	0.7742 (4)	0.0291 (4)	0.0451 (17)	
H302	0.0538	0.7739	0.0535	0.054*	
C303	0.0632 (3)	0.7592 (5)	−0.0438 (4)	0.055 (2)	
H303	0.0383	0.7493	−0.0696	0.066*	
C304	0.0905 (3)	0.7588 (5)	−0.0795 (4)	0.063 (2)	
H304	0.0842	0.7472	−0.1291	0.075*	
C305	0.1264 (3)	0.7750 (5)	−0.0435 (4)	0.063 (2)	
H305	0.1448	0.7751	−0.0683	0.076*	
C306	0.1364 (2)	0.7917 (4)	0.0309 (4)	0.0477 (18)	
H306	0.1612	0.8039	0.0558	0.057*	
C307	0.10216 (19)	0.9046 (4)	0.1558 (4)	0.0377 (15)	
C308	0.0669 (2)	0.9158 (4)	0.1655 (4)	0.0453 (17)	
H308	0.0531	0.8769	0.1751	0.054*	
C309	0.0520 (3)	0.9853 (5)	0.1609 (5)	0.064 (2)	
H309	0.0284	0.9930	0.1681	0.077*	
C310	0.0719 (3)	1.0414 (5)	0.1460 (6)	0.080 (3)	
H310	0.0621	1.0878	0.1435	0.096*	
C311	0.1069 (3)	1.0301 (5)	0.1342 (6)	0.071 (3)	
H311	0.1203	1.0690	0.1233	0.085*	
C312	0.1217 (2)	0.9624 (4)	0.1386 (4)	0.0495 (18)	
H312	0.1450	0.9550	0.1299	0.059*	
C401	0.21967 (18)	0.7613 (4)	0.3329 (4)	0.0362 (14)	
C402	0.22359 (19)	0.7185 (4)	0.3947 (4)	0.0411 (15)	
H402	0.2030	0.7102	0.4127	0.049*	
C403	0.2585 (2)	0.6886 (5)	0.4288 (5)	0.061 (2)	
H403	0.2616	0.6604	0.4705	0.073*	
C404	0.2883 (2)	0.7001 (5)	0.4016 (6)	0.071 (3)	
H404	0.3117	0.6792	0.4244	0.085*	
C405	0.2841 (2)	0.7413 (5)	0.3419 (6)	0.069 (3)	
H405	0.3047	0.7491	0.3238	0.083*	
C406	0.2497 (2)	0.7725 (4)	0.3070 (5)	0.0516 (19)	
H406	0.2472	0.8012	0.2658	0.062*	
C407	0.17832 (16)	0.8864 (3)	0.3373 (3)	0.0305 (13)	
C408	0.17667 (19)	0.8850 (4)	0.4097 (4)	0.0403 (15)	
H408	0.1741	0.8413	0.4319	0.048*	
C409	0.1788 (2)	0.9477 (4)	0.4478 (4)	0.0500 (18)	
H409	0.1783	0.9465	0.4963	0.060*	
C410	0.1816 (2)	1.0119 (4)	0.4157 (5)	0.056 (2)	
H410	0.1823	1.0546	0.4417	0.068*	
C411	0.1834 (2)	1.0139 (4)	0.3462 (5)	0.0527 (19)	
H411	0.1855	1.0581	0.3246	0.063*	
C412	0.18208 (19)	0.9513 (4)	0.3065 (4)	0.0427 (16)	
H412	0.1838	0.9534	0.2587	0.051*	
O1	0.1679 (7)	0.4193 (12)	0.3071 (17)	0.102 (6)*	0.5
H1	0.1783	0.4272	0.2751	0.153*	0.5
C11	0.1643 (12)	0.3480 (15)	0.314 (2)	0.161 (15)*	0.5
H11A	0.1869	0.3292	0.3477	0.241*	0.5
H11B	0.1602	0.3252	0.2673	0.241*	0.5
H11C	0.1432	0.3384	0.3328	0.241*	0.5
O1A	0.1721 (9)	0.4273 (16)	0.3540 (17)	0.164 (11)*	0.5
H1A	0.1867	0.4008	0.3836	0.246*	0.5
C11A	0.1680 (13)	0.403 (2)	0.2844 (18)	0.126 (16)*	0.5
H11D	0.1637	0.4426	0.2511	0.189*	0.5
H11E	0.1468	0.3702	0.2705	0.189*	0.5
H11F	0.1905	0.3775	0.2829	0.189*	0.5
C12	0.0153 (9)	0.1878 (19)	0.240 (3)	0.143 (13)	0.5
Cl1	0.0000	0.2759 (3)	0.2500	0.136 (2)	
Cl2	0.0099 (3)	0.1265 (5)	0.2798 (6)	0.148 (4)	0.5

### (4-Diazo-1,1,3,3,-tetraphenyl-1,3λ4-diphosphabut-4-yl-κP1)iodido[methylenebis(diphenylphosphine)-κ2P,P'](phenylmethanimine-κN)iridium(III) iodide–triiodide–dichloromethane–iodine–methanol (2/2/1/1/2) (4) Atomic displacement parameters (Å^2^)

**Table:**

	*U*^11^	*U*^22^	*U*^33^	*U*^12^	*U*^13^	*U*^23^
Ir1	0.02273 (13)	0.02864 (14)	0.02459 (13)	−0.00078 (8)	0.00637 (9)	−0.00107 (8)
I1	0.0262 (2)	0.0431 (3)	0.0455 (2)	−0.00481 (17)	0.00787 (17)	−0.00686 (19)
I3	0.0753 (4)	0.0922 (5)	0.0786 (4)	−0.0076 (4)	0.0351 (4)	0.0119 (4)
I2	0.0637 (5)	0.0537 (5)	0.0638 (5)	−0.0096 (5)	0.0289 (3)	0.0017 (4)
I4	0.1132 (16)	0.0966 (15)	0.0474 (8)	−0.0265 (13)	0.0213 (8)	−0.0073 (10)
I5	0.0682 (13)	0.0416 (13)	0.0479 (9)	−0.0051 (12)	0.0232 (8)	−0.0029 (12)
I6	0.0571 (15)	0.0670 (19)	0.0634 (11)	−0.0164 (13)	0.0131 (9)	−0.0131 (12)
I2A	0.052 (3)	0.045 (4)	0.081 (6)	−0.003 (4)	0.026 (4)	0.019 (4)
I4A	0.1442 (19)	0.0694 (11)	0.0533 (9)	−0.0075 (11)	0.0484 (11)	−0.0041 (9)
I5A	0.095 (3)	0.0558 (19)	0.095 (4)	0.0038 (14)	0.059 (3)	0.0079 (14)
I6A	0.0633 (18)	0.083 (3)	0.112 (4)	0.0089 (16)	0.0100 (19)	0.030 (2)
P1	0.0299 (8)	0.0315 (8)	0.0352 (8)	0.0026 (6)	0.0098 (7)	−0.0034 (7)
P2	0.0325 (9)	0.0353 (9)	0.0392 (9)	−0.0002 (7)	0.0097 (7)	0.0080 (7)
P3	0.0290 (8)	0.0380 (9)	0.0242 (7)	−0.0001 (6)	0.0051 (6)	0.0023 (6)
P4	0.0239 (8)	0.0355 (8)	0.0272 (7)	−0.0018 (6)	0.0051 (6)	0.0028 (6)
N1	0.026 (3)	0.028 (3)	0.032 (3)	−0.001 (2)	0.006 (2)	0.000 (2)
N2	0.087 (5)	0.058 (4)	0.021 (3)	−0.022 (4)	0.013 (3)	−0.005 (3)
N3	0.042 (3)	0.048 (3)	0.039 (3)	−0.007 (3)	0.014 (3)	0.007 (3)
C1	0.036 (3)	0.031 (3)	0.031 (3)	0.001 (3)	0.009 (3)	0.001 (3)
C2	0.034 (4)	0.038 (4)	0.044 (4)	0.004 (3)	0.010 (3)	0.004 (3)
C3	0.028 (3)	0.044 (4)	0.030 (3)	−0.001 (3)	0.009 (2)	0.001 (3)
C4	0.035 (3)	0.031 (3)	0.040 (3)	0.000 (3)	0.008 (3)	0.000 (3)
C5	0.042 (4)	0.051 (4)	0.037 (4)	0.009 (3)	0.004 (3)	−0.009 (3)
C6	0.054 (5)	0.044 (5)	0.081 (6)	0.006 (4)	0.007 (4)	−0.022 (4)
C7	0.082 (8)	0.054 (6)	0.125 (10)	0.010 (5)	−0.005 (7)	−0.037 (6)
C8	0.102 (10)	0.099 (10)	0.110 (10)	0.038 (8)	−0.005 (8)	−0.068 (9)
C9	0.107 (10)	0.130 (12)	0.075 (7)	0.049 (9)	0.031 (7)	−0.035 (8)
C10	0.070 (6)	0.082 (6)	0.055 (5)	0.023 (5)	0.025 (4)	−0.012 (5)
C101	0.040 (4)	0.037 (4)	0.046 (4)	0.002 (3)	0.022 (3)	−0.006 (3)
C102	0.046 (4)	0.053 (4)	0.057 (5)	0.015 (3)	0.023 (4)	0.009 (4)
C103	0.073 (6)	0.073 (6)	0.060 (5)	0.021 (5)	0.039 (5)	0.026 (4)
C104	0.055 (5)	0.083 (6)	0.078 (6)	0.020 (5)	0.046 (5)	0.018 (5)
C105	0.045 (5)	0.077 (6)	0.082 (6)	0.016 (4)	0.022 (4)	−0.004 (5)
C106	0.043 (4)	0.059 (5)	0.054 (4)	0.005 (3)	0.020 (3)	−0.011 (4)
C107	0.038 (4)	0.035 (4)	0.047 (4)	0.005 (3)	0.010 (3)	−0.013 (3)
C108	0.049 (4)	0.047 (4)	0.043 (4)	0.000 (3)	0.007 (3)	−0.010 (3)
C109	0.046 (5)	0.082 (6)	0.054 (5)	0.003 (4)	0.003 (4)	−0.030 (5)
C110	0.047 (5)	0.065 (6)	0.083 (7)	−0.003 (4)	0.006 (4)	−0.041 (5)
C111	0.060 (6)	0.035 (4)	0.119 (9)	−0.004 (4)	0.024 (6)	−0.024 (5)
C112	0.045 (4)	0.034 (4)	0.073 (5)	0.004 (3)	0.010 (4)	−0.008 (4)
C201	0.039 (4)	0.041 (4)	0.044 (4)	0.003 (3)	0.004 (3)	0.013 (3)
C202	0.046 (4)	0.079 (6)	0.044 (4)	0.002 (4)	0.013 (3)	0.018 (4)
C203	0.067 (6)	0.096 (7)	0.046 (5)	0.002 (5)	0.009 (4)	0.019 (5)
C204	0.060 (6)	0.083 (7)	0.057 (5)	−0.002 (5)	0.000 (4)	0.030 (5)
C205	0.042 (5)	0.098 (7)	0.065 (6)	0.008 (5)	0.002 (4)	0.037 (5)
C206	0.043 (4)	0.076 (6)	0.057 (5)	0.008 (4)	0.012 (4)	0.023 (4)
C207	0.035 (4)	0.036 (4)	0.047 (4)	−0.003 (3)	0.008 (3)	0.008 (3)
C208	0.044 (4)	0.043 (4)	0.052 (4)	0.002 (3)	0.016 (3)	0.009 (3)
C209	0.033 (4)	0.061 (5)	0.072 (5)	−0.003 (3)	0.010 (4)	0.016 (4)
C210	0.050 (5)	0.049 (5)	0.086 (6)	−0.020 (4)	0.014 (4)	0.009 (4)
C211	0.070 (6)	0.033 (4)	0.115 (8)	−0.009 (4)	0.032 (6)	0.010 (5)
C212	0.053 (5)	0.038 (4)	0.094 (7)	0.002 (4)	0.021 (5)	0.016 (4)
C301	0.040 (4)	0.038 (4)	0.027 (3)	0.006 (3)	0.005 (3)	0.006 (3)
C302	0.046 (4)	0.051 (4)	0.033 (3)	−0.006 (3)	0.003 (3)	0.002 (3)
C303	0.060 (5)	0.065 (5)	0.032 (4)	−0.013 (4)	0.002 (4)	−0.002 (3)
C304	0.089 (7)	0.068 (6)	0.028 (4)	−0.015 (5)	0.012 (4)	−0.004 (4)
C305	0.080 (6)	0.080 (6)	0.034 (4)	0.005 (5)	0.021 (4)	0.000 (4)
C306	0.054 (5)	0.059 (5)	0.030 (3)	0.003 (4)	0.012 (3)	0.007 (3)
C307	0.041 (4)	0.033 (3)	0.036 (3)	0.005 (3)	0.006 (3)	0.006 (3)
C308	0.039 (4)	0.047 (4)	0.048 (4)	0.008 (3)	0.008 (3)	0.008 (3)
C309	0.055 (5)	0.060 (5)	0.078 (6)	0.023 (4)	0.017 (4)	0.011 (5)
C310	0.088 (8)	0.049 (5)	0.097 (8)	0.021 (5)	0.020 (6)	0.017 (5)
C311	0.079 (7)	0.042 (5)	0.084 (7)	−0.007 (4)	0.013 (5)	0.018 (4)
C312	0.046 (4)	0.042 (4)	0.055 (4)	0.000 (3)	0.006 (3)	0.010 (3)
C401	0.024 (3)	0.043 (4)	0.037 (3)	0.002 (3)	0.000 (3)	−0.001 (3)
C402	0.034 (4)	0.049 (4)	0.035 (3)	0.002 (3)	0.001 (3)	0.001 (3)
C403	0.048 (5)	0.062 (5)	0.056 (5)	0.005 (4)	−0.010 (4)	0.005 (4)
C404	0.036 (5)	0.076 (6)	0.086 (7)	0.012 (4)	−0.007 (4)	0.006 (5)
C405	0.032 (4)	0.087 (7)	0.090 (7)	0.008 (4)	0.021 (4)	0.009 (5)
C406	0.033 (4)	0.054 (4)	0.066 (5)	0.002 (3)	0.011 (3)	0.014 (4)
C407	0.023 (3)	0.031 (3)	0.034 (3)	−0.005 (2)	0.002 (2)	−0.001 (3)
C408	0.040 (4)	0.040 (4)	0.038 (3)	−0.007 (3)	0.005 (3)	0.001 (3)
C409	0.051 (4)	0.058 (5)	0.038 (4)	−0.002 (4)	0.007 (3)	−0.010 (3)
C410	0.054 (5)	0.048 (4)	0.060 (5)	−0.007 (4)	0.006 (4)	−0.018 (4)
C411	0.051 (5)	0.037 (4)	0.064 (5)	−0.009 (3)	0.007 (4)	−0.003 (4)
C412	0.038 (4)	0.046 (4)	0.039 (4)	−0.006 (3)	0.004 (3)	0.005 (3)
C12	0.09 (2)	0.13 (3)	0.23 (4)	0.001 (18)	0.07 (2)	0.02 (3)
Cl1	0.166 (6)	0.076 (3)	0.128 (4)	0.000	−0.020 (4)	0.000
Cl2	0.146 (8)	0.101 (5)	0.163 (9)	0.009 (5)	−0.012 (6)	0.021 (5)

### (4-Diazo-1,1,3,3,-tetraphenyl-1,3λ4-diphosphabut-4-yl-κP1)iodido[methylenebis(diphenylphosphine)-κ2P,P'](phenylmethanimine-κN)iridium(III) iodide–triiodide–dichloromethane–iodine–methanol (2/2/1/1/2) (4) Geometric parameters (Å, º)

**Table:**

Ir1—N1	2.107 (5)	C205—C206	1.392 (12)
Ir1—C1	2.150 (6)	C205—H205	0.9400
Ir1—P4	2.3241 (15)	C206—H206	0.9400
Ir1—P1	2.3468 (16)	C207—C208	1.381 (10)
Ir1—P3	2.3536 (16)	C207—C212	1.394 (10)
Ir1—I1	2.7206 (5)	C208—C209	1.394 (11)
I3—I3^i^	2.8121 (16)	C208—H208	0.9400
I4—I5	2.946 (5)	C209—C210	1.356 (12)
I5—I6	2.913 (7)	C209—H209	0.9400
I4A—I5A	2.917 (7)	C210—C211	1.350 (13)
I5A—I6A	2.876 (9)	C210—H210	0.9400
P1—C101	1.826 (7)	C211—C212	1.392 (12)
P1—C107	1.828 (7)	C211—H211	0.9400
P1—C2	1.831 (7)	C212—H212	0.9400
P2—C1	1.753 (6)	C301—C306	1.369 (10)
P2—C207	1.788 (7)	C301—C302	1.396 (10)
P2—C201	1.796 (7)	C302—C303	1.374 (11)
P2—C2	1.797 (7)	C302—H302	0.9400
P3—C307	1.813 (7)	C303—C304	1.381 (13)
P3—C301	1.820 (6)	C303—H303	0.9400
P3—C3	1.829 (6)	C304—C305	1.353 (14)
P4—C401	1.798 (7)	C304—H304	0.9400
P4—C407	1.816 (6)	C305—C306	1.407 (11)
P4—C3	1.823 (6)	C305—H305	0.9400
N1—C4	1.267 (8)	C306—H306	0.9400
N1—H1N	0.86 (2)	C307—C308	1.395 (10)
N2—N3	1.095 (9)	C307—C312	1.397 (10)
N3—C1	1.305 (9)	C308—C309	1.409 (11)
C2—H2A	0.9800	C308—H308	0.9400
C2—H2B	0.9800	C309—C310	1.364 (14)
C3—H3A	0.9800	C309—H309	0.9400
C3—H3B	0.9800	C310—C311	1.404 (15)
C4—C5	1.459 (9)	C310—H310	0.9400
C4—H4	0.9400	C311—C312	1.376 (12)
C5—C6	1.393 (12)	C311—H311	0.9400
C5—C10	1.394 (12)	C312—H312	0.9400
C6—C7	1.378 (14)	C401—C406	1.366 (10)
C6—H6	0.9400	C401—C402	1.406 (10)
C7—C8	1.36 (2)	C402—C403	1.395 (10)
C7—H7	0.9400	C402—H402	0.9400
C8—C9	1.33 (2)	C403—C404	1.376 (14)
C8—H8	0.9400	C403—H403	0.9400
C9—C10	1.389 (15)	C404—C405	1.355 (14)
C9—H9	0.9400	C404—H404	0.9400
C10—H10	0.9400	C405—C406	1.396 (12)
C101—C102	1.387 (10)	C405—H405	0.9400
C101—C106	1.396 (10)	C406—H406	0.9400
C102—C103	1.375 (11)	C407—C412	1.378 (9)
C102—H102	0.9400	C407—C408	1.412 (9)
C103—C104	1.394 (13)	C408—C409	1.374 (10)
C103—H103	0.9400	C408—H408	0.9400
C104—C105	1.357 (13)	C409—C410	1.369 (12)
C104—H104	0.9400	C409—H409	0.9400
C105—C106	1.380 (11)	C410—C411	1.358 (12)
C105—H105	0.9400	C410—H410	0.9400
C106—H106	0.9400	C411—C412	1.392 (11)
C107—C112	1.381 (10)	C411—H411	0.9400
C107—C108	1.406 (10)	C412—H412	0.9400
C108—C109	1.403 (11)	O1—C11	1.353 (19)
C108—H108	0.9400	O1—H1	0.8300
C109—C110	1.351 (14)	C11—H11A	0.9700
C109—H109	0.9400	C11—H11B	0.9700
C110—C111	1.365 (15)	C11—H11C	0.9700
C110—H110	0.9400	O1A—C11A	1.382 (19)
C111—C112	1.402 (12)	O1A—H1A	0.8300
C111—H111	0.9400	C11A—H11D	0.9700
C112—H112	0.9400	C11A—H11E	0.9700
C201—C206	1.386 (11)	C11A—H11F	0.9700
C201—C202	1.397 (11)	C12—C12^ii^	1.30 (6)
C202—C203	1.379 (11)	C12—Cl2	1.42 (4)
C202—H202	0.9400	C12—Cl2^ii^	1.47 (3)
C203—C204	1.374 (14)	C12—Cl1	1.77 (4)
C203—H203	0.9400	Cl1—C12^ii^	1.77 (4)
C204—C205	1.349 (14)	Cl2—Cl2^ii^	1.174 (17)
C204—H204	0.9400	Cl2—C12^ii^	1.47 (3)
			
N1—Ir1—C1	86.8 (2)	C202—C203—H203	119.9
N1—Ir1—P4	87.44 (14)	C205—C204—C203	120.0 (8)
C1—Ir1—P4	100.39 (18)	C205—C204—H204	120.0
N1—Ir1—P1	170.93 (14)	C203—C204—H204	120.0
C1—Ir1—P1	84.37 (17)	C204—C205—C206	121.7 (8)
P4—Ir1—P1	96.31 (6)	C204—C205—H205	119.2
N1—Ir1—P3	90.39 (14)	C206—C205—H205	119.2
C1—Ir1—P3	170.90 (18)	C201—C206—C205	118.5 (8)
P4—Ir1—P3	70.80 (5)	C201—C206—H206	120.7
P1—Ir1—P3	98.63 (6)	C205—C206—H206	120.7
N1—Ir1—I1	84.97 (14)	C208—C207—C212	120.0 (7)
C1—Ir1—I1	91.96 (17)	C208—C207—P2	122.1 (5)
P4—Ir1—I1	165.12 (4)	C212—C207—P2	117.8 (6)
P1—Ir1—I1	93.11 (4)	C207—C208—C209	118.8 (7)
P3—Ir1—I1	96.44 (4)	C207—C208—H208	120.6
I6—I5—I4	174.7 (3)	C209—C208—H208	120.6
I6A—I5A—I4A	177.7 (3)	C210—C209—C208	120.8 (8)
C101—P1—C107	101.2 (3)	C210—C209—H209	119.6
C101—P1—C2	101.5 (3)	C208—C209—H209	119.6
C107—P1—C2	105.3 (3)	C211—C210—C209	120.9 (8)
C101—P1—Ir1	120.8 (2)	C211—C210—H210	119.6
C107—P1—Ir1	119.2 (2)	C209—C210—H210	119.6
C2—P1—Ir1	106.6 (2)	C210—C211—C212	120.4 (8)
C1—P2—C207	115.2 (3)	C210—C211—H211	119.8
C1—P2—C201	112.2 (3)	C212—C211—H211	119.8
C207—P2—C201	107.3 (3)	C211—C212—C207	119.1 (8)
C1—P2—C2	101.0 (3)	C211—C212—H212	120.4
C207—P2—C2	111.3 (3)	C207—C212—H212	120.4
C201—P2—C2	109.7 (3)	C306—C301—C302	120.5 (6)
C307—P3—C301	101.6 (3)	C306—C301—P3	119.9 (5)
C307—P3—C3	108.3 (3)	C302—C301—P3	119.3 (5)
C301—P3—C3	107.2 (3)	C303—C302—C301	119.2 (8)
C307—P3—Ir1	114.9 (2)	C303—C302—H302	120.4
C301—P3—Ir1	129.9 (2)	C301—C302—H302	120.4
C3—P3—Ir1	93.2 (2)	C302—C303—C304	120.4 (8)
C401—P4—C407	102.6 (3)	C302—C303—H303	119.8
C401—P4—C3	109.0 (3)	C304—C303—H303	119.8
C407—P4—C3	107.8 (3)	C305—C304—C303	120.4 (8)
C401—P4—Ir1	127.6 (2)	C305—C304—H304	119.8
C407—P4—Ir1	114.1 (2)	C303—C304—H304	119.8
C3—P4—Ir1	94.3 (2)	C304—C305—C306	120.4 (9)
C4—N1—Ir1	130.8 (4)	C304—C305—H305	119.8
C4—N1—H1N	116 (4)	C306—C305—H305	119.8
Ir1—N1—H1N	112 (4)	C301—C306—C305	119.0 (8)
N2—N3—C1	175.8 (7)	C301—C306—H306	120.5
N3—C1—P2	114.8 (5)	C305—C306—H306	120.5
N3—C1—Ir1	121.4 (5)	C308—C307—C312	119.5 (7)
P2—C1—Ir1	122.9 (3)	C308—C307—P3	119.7 (5)
P2—C2—P1	111.8 (3)	C312—C307—P3	120.8 (6)
P2—C2—H2A	109.3	C307—C308—C309	120.0 (7)
P1—C2—H2A	109.3	C307—C308—H308	120.0
P2—C2—H2B	109.3	C309—C308—H308	120.0
P1—C2—H2B	109.3	C310—C309—C308	119.7 (9)
H2A—C2—H2B	107.9	C310—C309—H309	120.1
P4—C3—P3	95.8 (3)	C308—C309—H309	120.1
P4—C3—H3A	112.6	C309—C310—C311	120.5 (9)
P3—C3—H3A	112.6	C309—C310—H310	119.8
P4—C3—H3B	112.6	C311—C310—H310	119.8
P3—C3—H3B	112.6	C312—C311—C310	120.2 (8)
H3A—C3—H3B	110.1	C312—C311—H311	119.9
N1—C4—C5	125.0 (6)	C310—C311—H311	119.9
N1—C4—H4	117.5	C311—C312—C307	120.1 (8)
C5—C4—H4	117.5	C311—C312—H312	120.0
C6—C5—C10	119.6 (8)	C307—C312—H312	120.0
C6—C5—C4	123.0 (7)	C406—C401—C402	119.9 (6)
C10—C5—C4	117.3 (8)	C406—C401—P4	120.8 (6)
C7—C6—C5	119.1 (11)	C402—C401—P4	119.2 (5)
C7—C6—H6	120.5	C403—C402—C401	118.9 (7)
C5—C6—H6	120.5	C403—C402—H402	120.5
C8—C7—C6	120.5 (12)	C401—C402—H402	120.5
C8—C7—H7	119.8	C404—C403—C402	120.3 (8)
C6—C7—H7	119.8	C404—C403—H403	119.8
C9—C8—C7	121.0 (10)	C402—C403—H403	119.8
C9—C8—H8	119.5	C405—C404—C403	120.1 (8)
C7—C8—H8	119.5	C405—C404—H404	120.0
C8—C9—C10	121.4 (13)	C403—C404—H404	120.0
C8—C9—H9	119.3	C404—C405—C406	121.0 (9)
C10—C9—H9	119.3	C404—C405—H405	119.5
C9—C10—C5	118.4 (11)	C406—C405—H405	119.5
C9—C10—H10	120.8	C401—C406—C405	119.8 (8)
C5—C10—H10	120.8	C401—C406—H406	120.1
C102—C101—C106	118.5 (6)	C405—C406—H406	120.1
C102—C101—P1	119.2 (5)	C412—C407—C408	118.6 (6)
C106—C101—P1	122.3 (5)	C412—C407—P4	123.7 (5)
C103—C102—C101	121.0 (8)	C408—C407—P4	117.7 (5)
C103—C102—H102	119.5	C409—C408—C407	119.9 (7)
C101—C102—H102	119.5	C409—C408—H408	120.1
C102—C103—C104	119.9 (8)	C407—C408—H408	120.1
C102—C103—H103	120.0	C410—C409—C408	120.7 (7)
C104—C103—H103	120.0	C410—C409—H409	119.6
C105—C104—C103	119.1 (7)	C408—C409—H409	119.6
C105—C104—H104	120.5	C411—C410—C409	119.9 (7)
C103—C104—H104	120.5	C411—C410—H410	120.0
C104—C105—C106	121.9 (8)	C409—C410—H410	120.0
C104—C105—H105	119.1	C410—C411—C412	120.9 (7)
C106—C105—H105	119.1	C410—C411—H411	119.5
C105—C106—C101	119.6 (8)	C412—C411—H411	119.5
C105—C106—H106	120.2	C407—C412—C411	119.9 (7)
C101—C106—H106	120.2	C407—C412—H412	120.0
C112—C107—C108	119.3 (7)	C411—C412—H412	120.0
C112—C107—P1	122.8 (6)	C11—O1—H1	109.3
C108—C107—P1	117.6 (5)	O1—C11—H11A	109.4
C109—C108—C107	119.6 (8)	O1—C11—H11B	109.7
C109—C108—H108	120.2	H11A—C11—H11B	109.5
C107—C108—H108	120.2	O1—C11—H11C	109.3
C110—C109—C108	120.9 (9)	H11A—C11—H11C	109.5
C110—C109—H109	119.6	H11B—C11—H11C	109.5
C108—C109—H109	119.6	C11A—O1A—H1A	109.6
C109—C110—C111	119.4 (8)	O1A—C11A—H11D	109.6
C109—C110—H110	120.3	O1A—C11A—H11E	109.4
C111—C110—H110	120.3	H11D—C11A—H11E	109.5
C110—C111—C112	122.2 (8)	O1A—C11A—H11F	109.4
C110—C111—H111	118.9	H11D—C11A—H11F	109.5
C112—C111—H111	118.9	H11E—C11A—H11F	109.5
C107—C112—C111	118.6 (8)	C12^ii^—C12—Cl2	65 (2)
C107—C112—H112	120.7	C12^ii^—C12—Cl2^ii^	61.6 (17)
C111—C112—H112	120.7	Cl2—C12—Cl2^ii^	47.9 (13)
C206—C201—C202	119.8 (7)	C12^ii^—C12—Cl1	68.4 (9)
C206—C201—P2	122.7 (6)	Cl2—C12—Cl1	126 (2)
C202—C201—P2	117.5 (6)	Cl2^ii^—C12—Cl1	123.6 (19)
C203—C202—C201	119.7 (8)	C12—Cl1—C12^ii^	43.1 (19)
C203—C202—H202	120.2	Cl2^ii^—Cl2—C12	67.9 (18)
C201—C202—H202	120.2	Cl2^ii^—Cl2—C12^ii^	64.2 (18)
C204—C203—C202	120.3 (9)	C12—Cl2—C12^ii^	54 (2)
C204—C203—H203	119.9		
			
C207—P2—C1—N3	97.2 (6)	C1—P2—C207—C212	173.0 (6)
C201—P2—C1—N3	−26.0 (6)	C201—P2—C207—C212	−61.2 (8)
C2—P2—C1—N3	−142.8 (5)	C2—P2—C207—C212	58.8 (7)
C207—P2—C1—Ir1	−93.5 (4)	C212—C207—C208—C209	−2.0 (12)
C201—P2—C1—Ir1	143.2 (4)	P2—C207—C208—C209	−177.4 (6)
C2—P2—C1—Ir1	26.5 (4)	C207—C208—C209—C210	0.5 (13)
C1—P2—C2—P1	−37.8 (4)	C208—C209—C210—C211	−0.2 (15)
C207—P2—C2—P1	85.0 (4)	C209—C210—C211—C212	1.3 (17)
C201—P2—C2—P1	−156.4 (4)	C210—C211—C212—C207	−2.7 (16)
C101—P1—C2—P2	162.8 (4)	C208—C207—C212—C211	3.0 (14)
C107—P1—C2—P2	−92.0 (4)	P2—C207—C212—C211	178.7 (8)
Ir1—P1—C2—P2	35.6 (4)	C307—P3—C301—C306	101.7 (6)
C401—P4—C3—P3	152.6 (3)	C3—P3—C301—C306	−11.8 (7)
C407—P4—C3—P3	−96.7 (3)	Ir1—P3—C301—C306	−121.4 (5)
Ir1—P4—C3—P3	20.3 (3)	C307—P3—C301—C302	−73.1 (6)
C307—P3—C3—P4	97.6 (3)	C3—P3—C301—C302	173.4 (6)
C301—P3—C3—P4	−153.6 (3)	Ir1—P3—C301—C302	63.8 (6)
Ir1—P3—C3—P4	−20.0 (3)	C306—C301—C302—C303	1.4 (11)
Ir1—N1—C4—C5	173.5 (5)	P3—C301—C302—C303	176.2 (6)
N1—C4—C5—C6	11.2 (11)	C301—C302—C303—C304	0.7 (12)
N1—C4—C5—C10	−166.2 (7)	C302—C303—C304—C305	−1.8 (14)
C10—C5—C6—C7	2.1 (13)	C303—C304—C305—C306	0.7 (14)
C4—C5—C6—C7	−175.3 (8)	C302—C301—C306—C305	−2.4 (11)
C5—C6—C7—C8	−1.0 (16)	P3—C301—C306—C305	−177.2 (6)
C6—C7—C8—C9	0 (2)	C304—C305—C306—C301	1.4 (13)
C7—C8—C9—C10	−1 (2)	C301—P3—C307—C308	97.5 (6)
C8—C9—C10—C5	1.9 (18)	C3—P3—C307—C308	−149.8 (5)
C6—C5—C10—C9	−2.5 (13)	Ir1—P3—C307—C308	−47.1 (6)
C4—C5—C10—C9	175.0 (8)	C301—P3—C307—C312	−79.7 (6)
C107—P1—C101—C102	47.5 (7)	C3—P3—C307—C312	33.0 (7)
C2—P1—C101—C102	155.9 (6)	Ir1—P3—C307—C312	135.6 (5)
Ir1—P1—C101—C102	−86.7 (6)	C312—C307—C308—C309	−2.7 (11)
C107—P1—C101—C106	−132.1 (6)	P3—C307—C308—C309	−179.9 (6)
C2—P1—C101—C106	−23.7 (7)	C307—C308—C309—C310	0.9 (13)
Ir1—P1—C101—C106	93.7 (6)	C308—C309—C310—C311	0.9 (16)
C106—C101—C102—C103	−1.2 (12)	C309—C310—C311—C312	−0.8 (16)
P1—C101—C102—C103	179.2 (7)	C310—C311—C312—C307	−1.0 (14)
C101—C102—C103—C104	−0.7 (14)	C308—C307—C312—C311	2.7 (12)
C102—C103—C104—C105	2.2 (15)	P3—C307—C312—C311	180.0 (7)
C103—C104—C105—C106	−1.9 (16)	C407—P4—C401—C406	−89.6 (7)
C104—C105—C106—C101	−0.1 (14)	C3—P4—C401—C406	24.6 (7)
C102—C101—C106—C105	1.6 (12)	Ir1—P4—C401—C406	136.1 (6)
P1—C101—C106—C105	−178.8 (7)	C407—P4—C401—C402	89.3 (6)
C101—P1—C107—C112	91.0 (7)	C3—P4—C401—C402	−156.5 (5)
C2—P1—C107—C112	−14.3 (7)	Ir1—P4—C401—C402	−45.0 (7)
Ir1—P1—C107—C112	−133.9 (6)	C406—C401—C402—C403	0.4 (11)
C101—P1—C107—C108	−82.3 (6)	P4—C401—C402—C403	−178.5 (6)
C2—P1—C107—C108	172.4 (6)	C401—C402—C403—C404	−0.9 (12)
Ir1—P1—C107—C108	52.9 (6)	C402—C403—C404—C405	0.9 (15)
C112—C107—C108—C109	−1.5 (11)	C403—C404—C405—C406	−0.3 (16)
P1—C107—C108—C109	172.0 (6)	C402—C401—C406—C405	0.2 (12)
C107—C108—C109—C110	1.0 (13)	P4—C401—C406—C405	179.1 (7)
C108—C109—C110—C111	0.3 (14)	C404—C405—C406—C401	−0.3 (15)
C109—C110—C111—C112	−1.2 (15)	C401—P4—C407—C412	108.3 (6)
C108—C107—C112—C111	0.7 (12)	C3—P4—C407—C412	−6.7 (6)
P1—C107—C112—C111	−172.5 (6)	Ir1—P4—C407—C412	−110.0 (5)
C110—C111—C112—C107	0.7 (14)	C401—P4—C407—C408	−72.0 (6)
C1—P2—C201—C206	−106.7 (7)	C3—P4—C407—C408	173.0 (5)
C207—P2—C201—C206	125.8 (7)	Ir1—P4—C407—C408	69.7 (5)
C2—P2—C201—C206	4.8 (8)	C412—C407—C408—C409	0.1 (10)
C1—P2—C201—C202	71.6 (7)	P4—C407—C408—C409	−179.6 (6)
C207—P2—C201—C202	−55.9 (7)	C407—C408—C409—C410	1.5 (11)
C2—P2—C201—C202	−176.9 (6)	C408—C409—C410—C411	−1.8 (13)
C206—C201—C202—C203	1.2 (13)	C409—C410—C411—C412	0.6 (13)
P2—C201—C202—C203	−177.2 (7)	C408—C407—C412—C411	−1.3 (10)
C201—C202—C203—C204	−0.7 (15)	P4—C407—C412—C411	178.4 (6)
C202—C203—C204—C205	0.1 (16)	C410—C411—C412—C407	1.0 (11)
C203—C204—C205—C206	0.1 (17)	Cl2—C12—Cl1—C12^ii^	−30.7 (15)
C202—C201—C206—C205	−1.0 (13)	Cl2^ii^—C12—Cl1—C12^ii^	28.6 (14)
P2—C201—C206—C205	177.2 (7)	C12^ii^—C12—Cl2—Cl2^ii^	73 (3)
C204—C205—C206—C201	0.4 (15)	Cl1—C12—Cl2—Cl2^ii^	105 (3)
C1—P2—C207—C208	−11.5 (7)	Cl2^ii^—C12—Cl2—C12^ii^	−73 (3)
C201—P2—C207—C208	114.3 (6)	Cl1—C12—Cl2—C12^ii^	31.6 (14)
C2—P2—C207—C208	−125.7 (6)		

Symmetry codes: (i) −*x*+1/2, −*y*+3/2, −*z*; (ii) −*x*, *y*, −*z*+1/2.

### (4-Diazo-1,1,3,3,-tetraphenyl-1,3λ4-diphosphabut-4-yl-κP1)iodido[methylenebis(diphenylphosphine)-κ2P,P'](phenylmethanimine-κN)iridium(III) iodide–triiodide–dichloromethane–iodine–methanol (2/2/1/1/2) (4) Hydrogen-bond geometry (Å, º)

*Cg* is the centroid of the C407–C412 ring.

**Table:**

*D*—H···*A*	*D*—H	H···*A*	*D*···*A*	*D*—H···*A*
N1—H1*N*···*Cg*	0.86 (2)	2.87 (5)	3.608 (6)	145 (4)
C408—H408···N2	0.94	2.57	3.35 (1)	141
C4—H4···I1	0.94	2.98	3.45 (1)	112
C2—H2*A*···O1	0.98	2.25	3.19 (2)	160
C2—H2*A*···O1*A*	0.98	2.28	3.11 (3)	142
C112—H112···O1	0.94	2.55	3.41 (3)	154
C212—H212···O1*A*	0.94	2.31	3.20 (4)	159
C3—H3*B*···I2	0.98	3.02	3.89 (1)	149
C106—H106···I2*A*^iii^	0.94	2.97	3.51 (1)	117
C205—H205···*Cg*^iv^	0.94	2.86	3.749 (9)	159

Symmetry codes: (iii) *x*, −*y*−1, *z*−1/2; (iv) −*x*+1/2, −*y*+3/2, −*z*+1.
